# Current developments on polyhydroxyalkanoates synthesis by using halophiles as a promising cell factory

**DOI:** 10.1186/s12934-020-01342-z

**Published:** 2020-04-07

**Authors:** Ruchira Mitra, Tong Xu, Hua Xiang, Jing Han

**Affiliations:** 1 State Key Laboratory of Microbial Resources, Institute of Microbiology, Chinese Academy of Sciences, Beijing, 100101 People’s Republic of China; 2 International College, University of Chinese Academy of Sciences, Beijing, 100049 People’s Republic of China; 3 College of Life Science, University of Chinese Academy of Sciences, Beijing, 100049 People’s Republic of China

**Keywords:** Polyhydroxyalkanoates, Halophiles, Production improvement, Cost reduction, Novel PHA biosynthesis, Metabolic engineering

## Abstract

Plastic pollution is a severe threat to our environment which necessitates implementation of bioplastics to realize sustainable development for a green world. Polyhydroxyalkanoates (PHA) represent one of the potential candidates for these bioplastics. However, a major challenge faced by PHA is the high production cost which limits its commercial application. Halophiles are considered to be a promising cell factory for PHA synthesis due to its several unique characteristics including high salinity requirement preventing microbial contamination, high intracellular osmotic pressure allowing easy cell lysis for PHA recovery, and capability to utilize wide spectrum of low-cost substrates. Optimization of fermentation parameters has made it plausible to achieve large-scale production at low cost by using halophiles. Further deeper insights into halophiles have revealed the existence of diversified and even novel PHA synthetic pathways within different halophilic species that greatly affects PHA type. Thus, precise metabolic engineering of halophiles with the help of advanced tools and strategies have led to more efficient microbial cell factory for PHA production. This review is an endeavour to summarize the various research achievements in these areas which will help the readers to understand the current developments as well as the future efforts in PHA research.

## Background

The petrochemical-derived plastics feature one of the most important components in our daily lives. However, with skyrocketing plastic production and usage, plastic pollution has become one of the most alarming and obvious threat to all forms of life [1]. Despite of its enormous societal benefits, plastics account for significant waste accumulation which mostly remains undegraded and recalcitrant for decades in our ecosystem [2]. A possible panacea to these problems may be the use of biodegradable and biosynthetic plastics as an alternative. Polyhydroxyalkanoates (PHA) are linear polyesters synthesized by a variety of microorganisms, serving as an intracellular reservoir for energy and carbon supply [3, 4]. Owing to their combined properties of biodegradability, biocompatibility, and thermoplasticity, PHA has gained substantial importance as a promising candidate for bio-based plastics as well as biomaterials [5]. Interestingly, properties of PHA are highly tuneable and versatile as they vary depending on the type and amount of monomer constituents. Till now, over 150 different monomers have been incorporated into PHA chains under various fermentation conditions, yielding PHA with different characteristics [6]. PHA has tremendous market potential but the high production cost has limited its commercial applications [7]. Recently, halophiles are attracting considerable interest as a promising and cost-effective producer of PHA [8].

Halophiles represent a distinctive and diversified group of microorganisms that have the ability to survive in hypersaline habitats such as saline lakes, salt pans, and salt marshes. They are widely spread in all the three domains of life, *Bacteria*, *Archaea*, and *Eukarya* [9]. Based on their optimum salinity for growth, microorganisms requiring salts are classified as mild, moderate, and extreme halophiles. Mild halophiles grow at a salt concentration of 1–6% (w/v) whereas moderate and extreme halophiles can grow at salt concentrations of 7–15% (w/v) and higher than 15% (w/v), respectively [10]. As a survival strategy, halophiles maintain a high osmotic pressure to adapt to the surrounding hypersaline environment by accumulating either high concentrations of K^+^ salts or soluble organic solutes (known as compatible solutes) [11]. Some compatible solutes have captured the interest of researchers due to their high value-added property. For instance, ectoine has high commercial importance as an osmostress protectant in cosmetic industry [12]. Additionally, hydrolytic enzymes including amylase, cellulase, protease, and xylanase, secreted by halophiles are also very useful for industrial processes under hypersaline conditions [13]. Furthermore, halophiles are a potent source of biosurfactant, bioemulsifiers, and various other chemicals including aminophenoxazinones, carotenoids, and bacteriorhodopsin [8, 14]. Especially, most halophiles have the inherent ability to accumulate PHA intracellularly.

The adaptation of halophiles to extreme conditions has bestowed them with unique potentials and advantages for PHA production. The foremost advantage is that the high salinity requirement reduces the chances of microbial contamination to a great extent [15]. PHA recovery cost is further reduced as cells can be easily lysed in normal water due to the high intracellular osmotic pressure [16]. Moreover, compared to the non-halophilic microbes, halophiles have the ability to produce PHA by utilizing various inexpensive raw materials, thus reducing the fermentation cost [17]. Thus, they are considered as a promising candidate for PHA production. The company named Bluepha in China is producing polyhydroxybutyrate (PHB) and poly(3-hydroxybutyrate-*co*-3-hydroxyvalerate) (PHBV) using a *Halomonas* species as microbial cell factory. However, there are still some challenges when using halophiles for large-scale PHA production. For example, treatment of the saline fermentation effluent is difficult [18]. The high salt concentration of medium corrodes fermentation equipment. Additionally, lack of genetic engineering tools and less well-defined systems might be also great challenges for large-scale production of PHA using halophiles. With growing research, strategies have been developed to overcome these issues. Saline wastewater can be treated by marine bacteria [19]. Salt-resistant fermentation equipments made of plastics, ceramics or carbon steel have been used to culture halophiles [8]. Especially, development of genetically engineered halophilic strains is likely to enhance the efficiency of large-scale PHA production by halophiles.

**Fig. 1:**
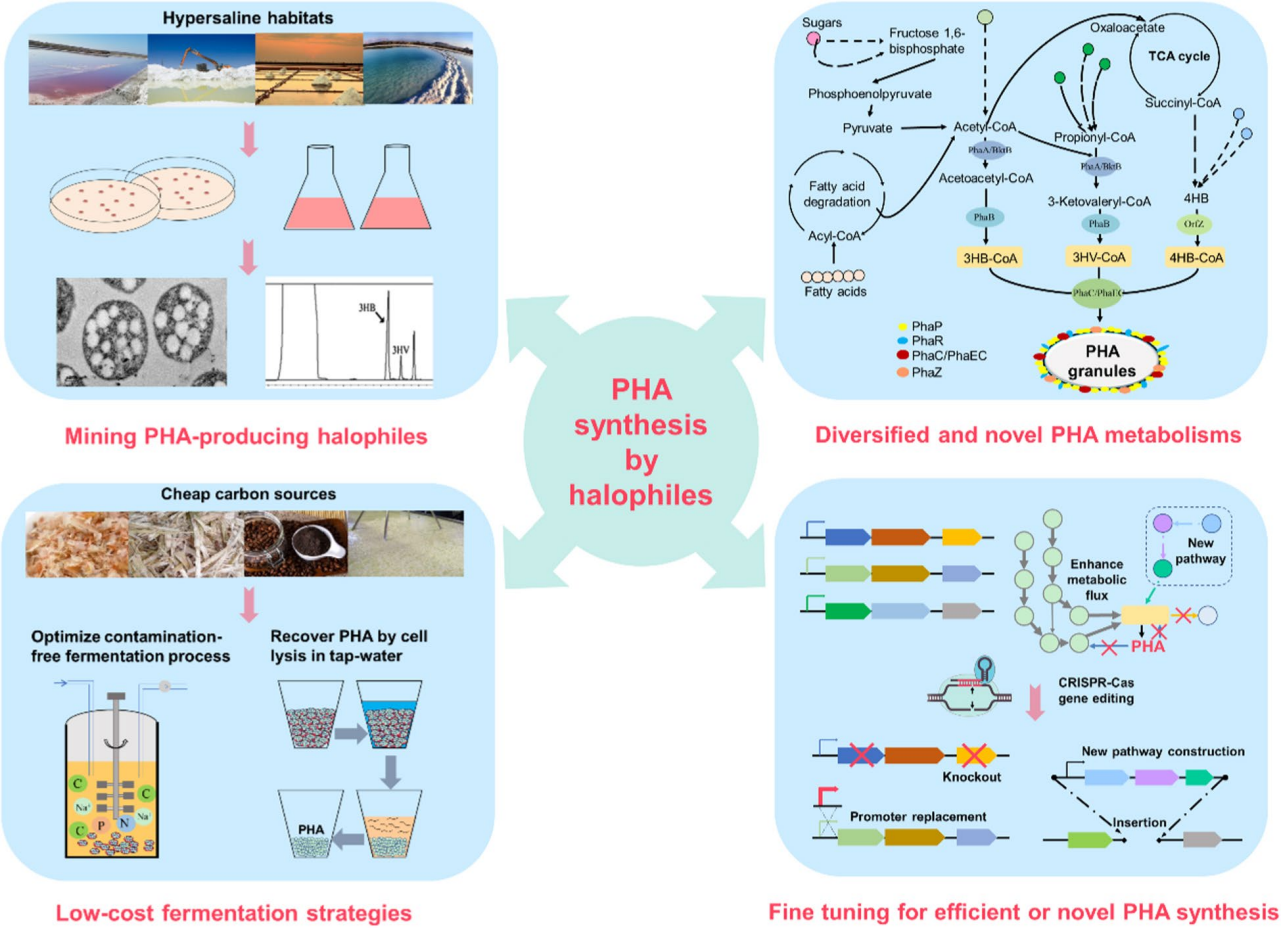
Summary of the PHA research achievements in Haloarchaea and Halophilic bacteria. Halophiles with strong PHA-accumulation ability are mined from various hypersaline habitats. PHA production cost is closely related with substrate usage, fermentation process, and PHA recovery. Halophiles are able to utilize a wide-range of low-cost substrates. PHA recovery from halophiles can be easily done by cell lysis using tap-water. The physicochemical parameters and fermentation modes have been optimized to enhance PHA production in halophiles. With the aim of genome sequencing technology and genetic manipulation tools, various PHA related genes, enzymes and pathways have been identified. With the help of advanced metabolic engineering tools and strategies, more efficient microbial cell factory has been developed for PHA production using halophiles

At present, there are many reviews already published on PHA synthesis, and its related challenges and future applications [15, 20–22]. However, most of these reviews focus on non-halophiles. An integrated review mainly presenting the current scenario of PHA synthesis by both the haloarchaea and halophilic bacteria is relatively less. Research on PHA synthesis by halophiles is accelerating and thus, it is urgent to present an updated and in-depth review on this field. In this review, the progresses on PHA synthesis by haloarchaea and halophilic bacteria have been outlined (Fig. 1). The first two parts of the review include significance of halophiles as PHA producer and the various fermentation strategies adopted to realize low cost halophile-mediated PHA production. The third part presents important insights on the PHA metabolism and its regulation in halophiles. Finally, the fourth part of the review deals with the application of metabolic engineering techniques for enhancement of PHA production and synthesis of novel PHA.

## Halophiles as a promising platform for PHA production

**Fig. 2:**
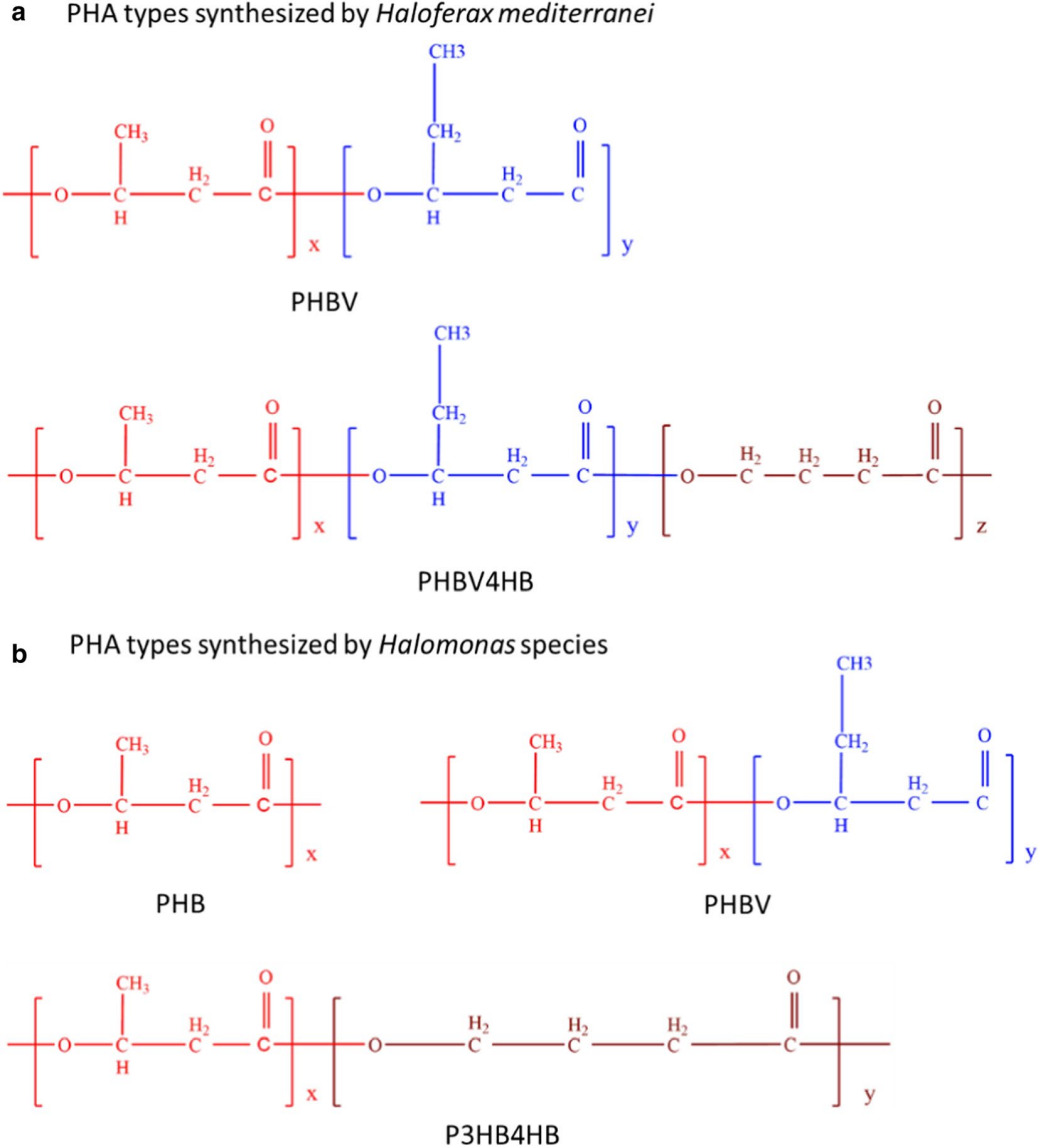
The PHA types synthesized by *Haloferax mediterranei* and *Halomonas* species. *Hfx. mediterranei* can synthesize PHBV and PHBV4HB. *Halomonas* species are able to accumulate PHB, PHBV, and P3HB4HB

Among halophiles, the haloarchaeon *Haloferax mediterranei* and the *Halomonas* species from bacterial domain have been extensively studied for efficient PHA production. Up to now, *Hfx. mediterranei* has been reported to synthesize PHBV and poly(3-hydroxybutyrate-*co*-3-hydroxyvalerate-*co*-4-hydroxybutyrate) (PHBV4HB) [23]; *Halomonas* species can accumulate PHB, PHBV, and poly(3-hydroxybutyrate-*co*-4-hydroxybutyrate) (P3HB4HB) [24–26] (Fig. 2). More and more promising candidates from different genera have already marked their prominence in this field. Thus, the following section attempts to introduce the wide range of PHA-producing halophiles to the readers.

### Haloarchaea as PHA producers

Ever since Kirk and Ginzburg reported the presence of PHB in *Halobacterium* sp. isolated from Dead Sea, (presently known as *Haloarcula marismortui*), more and more haloarchaeal species with PHA-accumulating ability have been identified (Table 1) [27]. In 1986, Fernandez-Castillo et al. detected the presence of PHB in four more haloarchaeal species which are now known as *Hfx. mediterranei*, *Haloferax volcanii*, *Haloferax gibbonsii*, and *Haloarcula hispanica* [28]. In the period from 1990 to 2000, PHA accumulation was detected in two more *Haloarcula* species, namely, *Haloarcula vallismortis* [29, 30] and *Haloarcula japonica* [30]. Following this, *Halopiger aswanensi* was found to accumulate PHB accounting about 53% of its cell dry weight (CDW), when n-butyric acid and sodium acetate were used as carbon sources [31, 32]. The presence of PHA granules was further observed in two *Haloquadratum walsbyi* species [33]. By 2010, PHA accumulation was reported in several other haloarchaeal species including, *Halostagnicola* sp., *Haloterrigena* sp., *Halobiforma* sp., *Haloarcula* sp., *Halobacterium* sp., *Halococcus* sp., *Halorubrum* sp., *Natrinema* sp. and haloalkaliphiles including *Natronobacterium* sp. and *Natronococcus* sp. [34–37]. In 2017, it was reported that *Halorubrum lacusprofundi,* the third most abundant species in the Deep Lake community of Antarctica, produced PHA-like granules at low temperature [38]. To date, haloarchaeal species belonging to almost 17 genera have been reported to exhibit PHA-accumulating ability (Table 1).

**Table 1:** Haloarchaeal genera with PHA-accumulating ability

Genus	Species	References
*Haloarcula*	*Haloarcula marismortui*	[27]
	*Haloarcula hispanica*	[28]
	*Haloarcula vallismortis*	[29, 30]
	*Haloarcula japonica*	[30]
	*Haloarcula amylolytica*	[35]
	*Haloarcula argentinensis*	[35]
*Haloferax*	*Haloferax mediterranei*	[28]
	*Haloferax volcanii*	[28]
	*Haloferax gibbonsii*	[28]
*Halopiger*	*Halopiger aswanensi*	[31, 32]
*Haloquadratum*	*Haloquadratum walsbyi*	[33]
*Halobacterium*	*Halobacterium noricense*	[36]
	*Halobacterium cutirubrum*	[35]
	*Halobacterium halobium*	[35]
*Halostagnicola*	*Halostagnicola larsenii*	[35]
*Haloterrigena*	*Haloterrigena hispanica*	[37]
	*Haloterrigena turkmenica*	[35]
*Halobiforma*	*Halobiforma nitratireducens*	[35]
	*Halobiforma haloterrestris*	[34]
*Halococcus*	*Halococcus morrhuae*	[35, 36]
	*Halococcus saccharolyticus*	[36]
	*Halococcus salifodinae*	[36]
	*Halococcus dombrowskii*	[36]
	*Halococcus hamelinensis*	[36]
	*Halococcus qingdaonensis*	[36]
*Halorubrum*	*Halorubrum coriense*	[36]
	*Halorubrum chaoviator*	[36]
	*Halorubrum litoreum*	[35]
	*Halorubrum trapanicum*	[35]
	*Halorubrum lacusprofundi*	[38]
*Halalkalicoccus*	*Halalkalicoccus tibetensis*	[35]
*Halogeometricum*	*Halogeometricum borinquense*	[42]
*Halogranum*	*Halogranum amylolyticum*	[46]
*Natrinema*	*Natrinema altunense*	[35]
	*Natrinema pallidum*	[35]
	*Natrinema pellirubrum*	[35]
	*Natrinema ajinwuensis*	[45]
*Natronobacterium*	*Natronobacterium gregoryi*	[35, 36]
*Natronorubrum*	*Natronorubrum tibetense*	[35]
*Natronococcus*	*Natronococcus occultus*	[36]

Among all the haloarchaeal strains, *Hfx mediterranei* is so far the most preferable PHA producer. It has several advantages such as high growth rate, metabolic versatility, genetic stability, and an efficient transformation system [14]. Many reports have also shown that *Hfx. mediterranei* has the ability to utilize various industrial and household waste products as carbon sources for synthesizing PHA with considerable amount of productivity. Moreover, detailed investigations have revealed that the PHA produced by the strain is a copolymer of 3-hydroxybutyrate (3HB) and 3-hydroxyvalerate (3HV) from unrelated carbon sources [39]. PHBV is a commercially more efficient and favourable polymer than PHB [4]. Most organisms require 3HV precursor for PHBV synthesis whereas *Hfx. mediterranei* can efficiently synthesize PHBV without any precursor, thus greatly reducing the production cost [40]. Moreover, it was clearly evident that the cost of PHA production using *Hfx. mediterranei* was much lower than other bacteria including *Alcaligenes eutrophus*, *Pseudomonas hydrogenovora*, and *Hydrogenophaga pseudoflava* [40]. Thus, *Hfx. mediterranei* is considered as one of the most promising microbial cell factories for large-scale PHBV production. Other strains including *Har. hispanica*, *Halogeometricum borinquense*, and *Natrinema* species were also found to accumulate PHBV only by utilizing unrelated carbon sources [41–44]. For example, *Natrinema ajinwuensis* accumulated PHBV containing 13.93 mol% 3HV by using glucose as the sole carbon source [45]. *Halogranum amylolyticum* could also efficiently accumulate PHBV with 20.1 mol% 3HV from glucose [46]. However, unlike *Hfx. mediterranei*, these haloarchaea have drawbacks as a platform to be further optimized for PHA production, such as low 3HV content in PHBV, slow growth rate, requirement for too high salinity, or absence of tractable genetic transformation system. Nevertheless, these reports indicate that with passing years more and more haloarchaea are being identified with PHBV synthesis capability.

### Halophilic bacteria as PHA producers

Within the bacterial domain, most PHA producers requiring salts belong to the family *Halomonadaceae.* Of almost 12 genera (as of May 2017) of *Halomonadaceae*, the genus *Halomonas* is known to accumulate short-chain-length PHA (Table 2). Most *Halomonas* sp. have lower NaCl requirement (3–15%) for optimal growth compared to haloarchaea but the concentration is sufficient enough to prevent microbial contamination. *Halomonas boliviensis*, an alkali tolerant and moderately halophilic bacterium, has the ability to efficiently synthesize PHB at an average content of 50% (wt) by utilizing glucose, sucrose, maltose, xylose as well as wheat bran [47–49]. In 2010, an alkaliphilic halophile, *Halomonas* sp. KM-1 was reported to produce PHB using waste glycerol as sole carbon source [50]. Another halophilic strain *Halomonas bluephagenesis* TD01 was cultured in a two-stage unsterile, open and continuous fermentation process yielding PHB up to 65–70% (wt) using glucose [51]. More and more *Halomonas* species have been found to synthesize PHB by using glucose or other cheap carbon sources (Table 2).

**Table 2:** Halophilic bacteria with PHA-accumulating ability

Genus	Species	References
*Halomonas*	*Halomonas boliviensis*	[47–49]
	*Halomonas* sp. KM-1	[50, 144]
	*Halomonas bluephagenesis* TD01	[51]
	*Halomonas nitroreducens*	[145]
	*Halomonas* sp. O-1	[101]
	*Halomonas elongata*	[101]
	*Halomonas halophila*	[73]
	*Halomonas marina*	[53]
	*Halomonas maura*	[146]
	*Halomonas ventosae*	[147]
	*Halomonas halodenitrificans*	[148]
	*Halomonas halodeneurihalina*	[148]
	*Halomonas salina*	[148]
	*Halomonas* sp. SF2003	[102]
	*Halomonas profundu*s	[54]
	*Halomonas campisalis*	[56]
	*Halomonas hydrothermalis*	[77]
*Vibrio*	*Vibrio proteolyticus*	[55]
*Yangia*	*Yangia* sp. ND199	[57]
	*Yangia* sp. CCB-MM3	[107]
*Paracoccus*	*Paracoccus* sp. LL1	[75]

However, unlike *Hfx. mediterranei*, most halophilic bacteria require 3HV precursor to produce PHBV. *H. bluephagenesis* TD01 produces PHBV when propionic acid or valeric acid is provided additionally with glucose [52]. *Halomonas marina* produces PHBV with 12.8 mol% 3HV in glucose medium supplemented with valeric acid [53]. Similarly, *Halomonas profundu*s produces PHBV with different molar fractions of 3HV in the presence of valeric acid and propionic acid [54]. Very recently, a PHB-producing halophile named *Vibrio proteolyticus* was isolated from Korean seas and it was additionally found to produce PHBV containing 15.8 mol% 3HV when propionic acid was added as a co-substrate of fructose [55]. As an exception, *Halomonas campisalis*, a moderately haloalkalitolerant bacterium, was reported to produce PHBV with 3.6 mol% 3HV from maltose [56]. Although the 3HV content in this copolymer was quite low, this was a significant finding as it was the first report on PHBV synthesis by a halophilic bacterium using unrelated carbon source. In 2015, *Yangia* sp. ND199, belonging to the *Rhodobacteraceae* family, was reported to accumulate PHBV with a range of 1–7 mol% 3HV from unrelated carbon source including glucose, maltose, sucrose and glycerol [57]. Taken together, although *H. bluephagenesis* TD01 synthesize only PHB from unrelated carbon source, it has emerged as a strong candidate for industrial PHA production with the establishment of tractable genetic system, the completion of genome sequencing, and the wide application of metabolic engineering.

## Fermentation strategies for reducing the cost of halophile-mediated PHA production

The large-scale production of PHA has high benefits in fermentation industry. A major drawback of PHA production is its economical uncompetitiveness with petroleum-based plastics. However, it has been possible to employ non-halophiles including *Alcaligenes latus*, *Bhurkolderia* sp., *Ralstonia eutropha*, and recombinant *Escherichia coli* for industrial PHA production after numerous attempts of cost reduction [58]. It is obvious that commercialization of halophiles will have added benefits of cost-effective and contamination-free fermentation compared to these non-halophiles. Three important factors contribute to the cost of PHA production-substrate usage, fermentation process, and PHA recovery. As PHA recovery from halophiles can be easily done by cell lysis using tap-water, rigorous efforts have been devoted to the first two factors. PHA synthesized by various halophiles using cheap carbon sources are summarized in Tables 3 and 4.

**Table 3:** PHA production by haloarchaea using various substrates

Microorganism	Carbon source	PHA type	PHA production	Fermentation operation mode	References
*Har. hispanica*	Glucose	P(3HB-*co*-3.18 mol% 3HV)	Content of 12.26% (wt)	Shake flasks	[105]
*Har. marismortui*	100% pre-treated vinasse	PHB	Content of 30% (wt); yield of 0.77 g/g; productivity of 0.02 g/L/h	Shake flasks	[69]
*Hfx. mediterranei*	Glucose	P(3HB-*co*-12.4 mol% 3HV)	Content of 16.4% (wt)	Shake flasks	[35]
*Hfx. mediterranei*	Extruded starch	P(3HB-*co*-10.4 mol% 3HV)	Content of 50.8% (wt)	Fed-batch	[85]
*Hfx. mediterranei*	25% pre-treated vinasse	P(3HB-*co*-12.36 mol% 3HV)	Content of 70% (wt); yield of 0.87 g/g; productivity of 0.21 g/L/h	Shake flasks	[61]
*Hfx. mediterranei*	50% pre-treated vinasse	P(3HB-*co*-14.09 mol% 3HV)	Content of 66% (wt); yield of 0.52 g/g; productivity of 0.18 g/L/h	Shake flasks	[61]
*Hfx. mediterranei*	Olive mill wastewater	P(3HB-*co*-6.5 mol% 3HV)	Content of 43% (wt)	Shake flasks	[62]
*Hfx. mediterranei*	Enzymatic hydrolysate of cheese whey	P(3HB-*co*-6 mol% 3HV)	Content of 72.8% (wt), yield of 0.29 g/g; productivity of 0.09 g/L/h	42-L bioreactor	[23]
*Hfx. mediterranei*	Chemical hydrolysate of cheese whey	P(3HB-*co*-1.5 mol% 3HV)	Content of 53% (wt); yield of 0.78 g/g; productivity of 4.04 g/L/day	2-L bioreactor	[64]
*Hfx. mediterranei*	Waste stillage	P(3HB-*co*-15.4 mol% 3HV)	Content of 71% (wt); yield of 0.35 g/g; productivity of 0.17 g/L/h	Shake flasks	[65]
*Hfx. mediterranei*	Waste stillage	P(3HB-*co*-17.9 mol% 3HV)	Content of 63% (wt); yield of 0.27 g/g; productivity of 0.14 g/L/h	Plug-flow reactor	[66]
*Hfx. mediterranei*	Crude glycerol	P(3HB-*co*-10 mol% 3HV)	Content of 76% (wt); yield of 0.19 g/g; productivity of 0.12 g/L/h	10-L bioreactor	[68]
*Hfx. mediterranei*	*Ulva* sp. hydrolysate	P(3HB-*co*-8 mol% 3HV)	Content of 55% (wt); productivity of 0.035 g/L/h	Duran bottle	[71]
*Hfx. mediterranei*	Whey sugars, sodium valerate and γ-butyrolactone	P(3HB-*co*-21.8 mol% 3HV-*co*-5.1 mo% 4HB)	Content of 87.5% (wt); yield of 0.20 g/g; productivity of 0.14 g/L/h	10-L bioreactor	[23]
*Hfx. mediterranei*	Crude glycerol phase and γ-butyrolactone	P(3HB-*co*-10.0 mol% 3HV-*co*-5.0 mol% 4HB)	Content of 68.5% (wt); yield of 0.16 g/g; productivity of 0.10 g/L/h	10-L bioreactor	[68]
*Hgn. amylolyticum*	Glucose	P(3HB-*co*-20.1 mol% 3HV)	Content of 26.1% (wt)	Shake flasks	[46]
*Hgn. amylolyticum*	Starch	P(3HB-*co*-18.7 mol% 3HV)	Content of 16.2% (wt)	Shake flasks	[46]
*Hgn. amylolyticum*	Glycerol	P(3HB-*co*-19.6 mol% 3HV)	Content of 7.2% (wt)	Shake flasks	[46]
*Hgm. borinquense*	Sugarcane bagasse hydrolysate	P(3HB-*co*-13.29 mol% 3HV)	Content of 45.7% (wt); yield of 0.253 g/g; productivity of 0.0113 g/L/h	Shake flasks	[43]
*Nnm. ajinwuensis*	Glucose	P(3HB-*co*-13.93 mol% 3HV)	Content of 61.02% (wt); productivity of 0.21 g/L/h	Repeated shake flasks	[45]

**Table 4:** PHA production by halophilic bacteria using various substrates

Microorganism	Carbon source	PHA type	PHA content (% CDW)	Fermentation operation mode	References
*H. halphila*	Glucose	PHB	81.5	Shake flasks	[73]
*H. halphila*	Cellobiose	PHB	90.8	Shake flasks	[73]
*H. halphila*	Spent coffee grounds	PHB	61.95	Shake flasks	[73]
*H. halphila*	Molasses	PHB	64.06	Shake flasks	[73]
*H. halphila*	Cheese whey hydrolysate	PHB	38.32	Shake flasks	[73]
*H. halphila*	Sawdust hydrolysates	PHB	46.85	Shake flasks	[73]
*H. halphila*	Diluted corn stover hydrolysate	PHB	38.67	Shake flasks	[73]
*H. boliviensis*	Maltose	PHB	58.8	Shake flasks	[48]
*H. boliviensis*	Starch hydrolysate	PHB	35–49	2-L fermentor	[48]
*H. boliviensis*	Butyric acid and sodium acetate	PHB	54	Shake flasks	[47]
*H. boliviensis*	Butyric acid and sodium acetate	PHB	88	2-L fermentor	[47]
*H. boliviensis*	Glucose or sucrose	PHB	~ 55	2-L fermentor	[47]
*H. boliviensis*	Wheat bran and digested potato waste	PHB	43	2-L fermentor	[49]
*H. boliviensis*	Volatile fatty acid (VFA)	P(3HB-*co*-8.5 mol% 3HV)	70	2-L bioreactor	[74]
*Halomonas* sp. KM-1	3% Waste glycerol	PHB	39	Shake flasks	[50]
*H. marina*	Glucose	PHB	> 59	Shake-flasks	[53]
*H. marina*	Glucose and 0.1% (w/v) valerate	P(3HB-*co*-12.8 mol% 3HV)	80	Shake-flasks	[53]
*V. proteolyticus*	Fructose	PHB	54.7	Shake flasks	[55]
*V. proteolyticus*	Fructose and 0.3% propionic acid	P(3HB-*co*-15.8 mol% 3HV)	≈ 30	Shake flasks	[55]
*H. hydrothermalis*	*Jatropha* biodiesel byproduct	PHB	75	Shake flasks	[77]
*H. hydrothermalis*	*Jatropha* biodiesel byproduct and 0.35% SDCLA	P(3HB-*co*-81 mol% 3HV)	73.3	Shake flasks	[78]
*H. campisalis*	Maltose	P(3HB-*co*-3.6 mol% 3HV)	45–81	Shake flasks	[56]
*Paracoccus* sp. LL1	Glucose	PHB	62	Shake flasks	[75]
*Paracoccus* sp. LL1	Xylose	PHB	59	Shake flasks	[75]
*Paracoccus* sp. LL1	Cellobiose	PHB	59	Shake flasks	[75]
*Paracoccus* sp. LL1	Corn stover hydrolysate	PHB	72.4	5-L fermentor	[75]
*Yangia* sp. ND199	Glycerol	P(3HB-*co*-2.9 mol% 3HV)	52.8	Shake flasks	[57]
*H. bluephagenesis* TD01	Glucose	PHB	65–70	Open fed-batch and continuous	[51]
*H. campaniensis*	Mixed substrate similar to kitchen waste	PHB	70	Open fed-continuous	[95]

### Low-cost substrate usage by haloarchaea

Besides the common challenges faced by halophiles, the major bottleneck faced by haloarchaea species, such as *Hfx. mediterranei*, is the low PHA productivity resulted from slow growth rate, and failing to achieve high cell-density cultivation and continuous fermentation. Due to this problem, *Hfx. mediterranei* has not been used to produce PHA in a large scale despite the fact that it does not require additional 3HV precursor. A possible strategy to overcome these challenges is usage of low-cost substrate which can compensate the high production cost resulting from low productivity. The raw materials account for almost 40–48% of the PHA production cost [59]. Replacement of the pure and expensive raw materials for PHA production is a big challenge for the researchers.

*Hfx. mediterranei* have the ability to utilize cheap carbon source and even agro-industrial wastes to synthesize PHA (Table 3). Utilization of waste by-products not only helps in waste management but also contributes to reducing PHA production cost. For instance, ethanol production from sugarcane or sugar beet generates considerable amounts of vinasse, which if indiscriminately disposed can be a potential risk to the eco-system [60]. Interestingly, *Hfx. mediterranei* can utilize pre-treated vinasse and accumulate 70% (wt) PHBV [61]. Olive mill wastewater, a highly problematic waste generated from olive oil industry, serves as the sole carbon source for *Hfx. mediterranei*, yielding 43% (wt) of PHBV [62]. Chitin is one of the most abundant natural polysaccharides. Seafood industries generate enormous amounts of chitin waste which are mostly recalcitrant in nature. *Hfx. mediterranei* encodes the enzymes of chitin catabolism and can effectively utilize chitin as a sole carbon source to produce PHBV at a concentration of 1 g/L [63]. Cheese whey, the major by-product of dairy industry is an enriched source of milk nutrients. Since, *Hfx. mediterranei* was unable to utilize lactose, enzymatically hydrolysed cheese whey was used as the sole carbon source, and 72.8% (wt) of PHBV was produced [23]. Because enzymatic hydrolysis can incur additional cost, chemical hydrolysis using hydrochloric acid was further employed to hydrolyse whey into glucose and galactose [64]. Micronutrients were additionally supplemented to favour galactose utilization for PHBV synthesis after glucose was consumed, which led to production of 53% (wt) PHBV in *Hfx. mediterranei*. Stillage emanating from rice-based ethanol manufacturing unit is an attractive low-cost substrate for PHA production. *Hfx. mediterranei* utilized raw stillage and produced 71% (wt) PHBV [65]. When stillage was used to produce PHBV in a plug-flow reactor configuration of the activated sludge process, *Hfx. mediterranei* was able to accumulate 63% (wt) PHBV while 99.3% of the medium salt was recovered for re-use [66]. Additionally, the cost for 1890 tons of PHBV was estimated to be as low as US$ 2.05/kg. This was almost comparable with the production cost of 2000 tons PHB by *Cupriavidus necator* (US$2.58/kg) [67]. Thus, this study holds high significance for constructing pilot-scale plant for PHBV production using *Hfx. mediterranei* and also forms a basis for integration of PHA production with ethanol manufacturing unit to minimize the overall cost. Crude glycerol is the waste by-product of biodiesel production. Interestingly, *Hfx. mediterranei* could utilize crude glycerol to give a PHBV concentration (16.2 g/L) similar to that produced from pure glycerol (13.4 g/L) [68]. Thus, utilization of crude glycerol opens up a possibility to integrate PHA production with the existing biodiesel production. This will significantly save the operational and transportation cost.

Other haloarchaeon including *Har. marismortui* and *Hgm. borinquense* strain E3 also have the ability to use low-cost substrates. *Har. marismortui* can utilize pre-treated vinasse to accumulate 30% (wt) PHB [69]. Sugarcane bagasse is a major lignocellulosic fibrous residue of sugarcane industry. *Hgm. borinquense* strain E3 showed 45.7% (wt) accumulation of PHBV when hydrolysates of sugarcane bagasse were utilized as a feedstock for PHA synthesis [43].

Other than the agro-industrial wastes, macroalgal biomass is a non-conventional yet promising feedstock for PHA production. Macroalgae has several advantages such as renewable, independent of land cultivation, and requirement of very less nutrition for growth [70]. *Ulva* sp., a green macroalgae, is a potential candidate for wastewater remediation. Interestingly, *Hfx. mediterranei* was capable to produce 55% (wt) PHBV by utilizing supercritical hydrolysate of *Ulva* sp. [71]. Thus, this is a double benefitted approach towards value-added polymer synthesis as well as waste remediation. Additionally, recycling of the waste streams emanating from PHA production unit is also a sustainable approach. Koller et al. investigated the possibility to recycle the spent fermentation broth during PHA synthesis by *Hfx. mediterranei* using the surplus whey from dairy industry [72]. Interestingly, a considerable part of the fresh PHA fermentation medium could be replaced with the spent fermentation broth. Furthermore, cell debris left after PHA production could replace almost 29% of the yeast extract in the fresh fermentation process. However, the salty spent fermentation broth might contain inhibitory metabolites which can adversely affect the growth of *Hfx. mediterranei*, although, further experimentation and fine-tuning of the process can help to overcome the drawback.

### Low-cost substrate usage by halophilic bacteria

There are several halophilic bacteria which can efficiently utilize low-cost substrates for PHA production. Spent coffee grounds, the primary waste by-product of coffee processing industry, are still an unusual substrate for valorization. *Halomonas halophila* was capable of utilizing hydrolysates of spent coffee grounds to produce 61.95% (wt) of PHB [73]. Furthermore, *H. halphila* showed the ability to utilize molasses, cheese whey hydrolysates, sawdust hydrolysates, and twice diluted corn stover hydrolysate to produce up to 64% (wt) PHB. This indicated the robustness of *H. halphila* for PHA production by using inexpensive raw materials. Volatile fatty acids (VFA) are organic compounds that are produced in huge quantity during acidogenic fermentation of food waste or municipal solid waste. *H. boliviensis* inherently produces PHB, however, in presence of VFA mixture containing acetic acid, propionic acid, butyric acid, and valeric acid, it could generate PHBV with 17.5 mol% and 8.5 mol% 3HV fraction in shake flask experiments and 2-L bioreactor, respectively [74]. Wheat bran is a cheap and readily available agricultural residue that gives high potentials to commercial production of PHB. Additionally, anaerobic digest of potato waste is a potent source of acetic and butyric acid. Using wheat bran hydrolysate and digested potato waste, *H. boliviensis* accumulated 43% (wt) PHB [49]. In a study conducted by Sawant et al., pre-treated corn stover was enzymatically hydrolysed by crude cellulase obtained from co-culture of *Trichoderma reesei* and *Aspergillus niger* [75]. The obtained hydrolysate mainly contained glucose, xylose, and cellobiose. This hydrolysate was further used as a feedstock for *Paracoccus* sp. LL1 (a moderate halophile) and 72.4% (wt) of PHB was achieved. Interestingly, the PHA yield was higher than that obtained from the sugar mixture containing pure glucose, xylose, and cellobiose. *Jatropha curcas* is a potential biofuel plant due to the high amounts of oil in its seeds [76]. *Halomonas hydrothermalis* SM-P-3 M has the ability to utilize the crude glycerol generated during the biofuel production process, without any pre-treatment or pH adjustment, to produce PHB up to 75% (wt) [77]. Thus, *Jatropha* biodiesel by-product, which is otherwise difficult to handle for its impurity and huge quantity, emerged as a possible substrate for PHB production. This is an innovative strategy which can make both the biodiesel and PHA production process economically more feasible. In the subsequent study, the PHA production process was modified to produce PHBV [78]. κ-carrageenan, extracted from granular biomass of *Kappaphycus alvarezii*, was subjected to hydrolysis to obtain SDCLA (seaweed derived crude levulinic acid). 3HV molar fraction ranging from 50 to 80 mol% was incorporated into polymer chains when different concentrations of SDCLA were co-fed with the *Jatropha* biodiesel by-product. PHA production was inhibited to half when pure levulinic acid was co-fed which indicated that the composition of SDCLA has a vital effect on the PHA accumulation by *H. hydrothermalis*. Thus, application of SDCLA with biodiesel residue is an economical and practical approach towards production of PHA with high 3HV molar fraction.

### Effect of fermentation conditions on PHA synthesis in haloarchaea

Optimizing the fermentation conditions is another key to improve PHA production. Process parameters and substrate feeding strategies are important influencing factors for product synthesis. For instance, Cui et al. cultivated *Hfx. mediterranei* in synthetic molasses wastewater at different temperatures (15, 20, 25, and 35 °C), and found that large quantities of PHA were accumulated at 35 °C [79]. At high culture temperatures, *Hfx. mediterranei* undergoes temperature-driven fast metabolism which causes nitrogen deficiency and triggers PHA overproduction. Thus, the following sections are an overview showing how the physico-chemical parameters and fermentation modes can affect the PHA production in haloarchaea, both quantitatively and qualitatively.

### C/N ratio

*Hfx. mediterranei* is a well-researched haloarchaeon for PHA synthesis since 1980’s. Early investigations on *Hfx. mediterranei* have shown that phosphate limitation, nitrogen source, and abundant carbon supply are important factors for accumulating large amounts of PHB (later reconfirmed as PHBV) [28, 80]. These fermentation conditions were also found to be an influencing factor for 3HV content in PHBV. *Hfx. mediterranei* produces PHBV containing almost 10 mol% of 3HV inherently from glucose. Ferre-Guell et al. showed that it is possible to obtain PHBV with varying 3HV content by changing nitrogen source [81]. Ammonium salt enhanced the 3HV content in the polymer compared to nitrate salt. Moreover, the carbon/nitrogen (C/N) molar ratio also affected the 3HV molar fraction in a narrow range. For example, in the presence of ammonium salt, increasing the C/N molar ratio from 8 to 34 decreased the 3HV fraction from 16.9 to 11.7 mol%. With further increasing the C/N ratio to 42, no 3HV fraction was observed in PHA. Contrarily, for the nitrate salt, increasing the C/N ratio from 8 to 42, only slightly changed the 3HV fraction from 12.5 to 11.6 mol%. Thus, altering the nitrogen source and C/N ratio can be a helpful strategy to obtain PHBV polymer with different 3HV contents from *Hfx. mediterranei.* A major drawback that lowers the efficiency of *Hfx. mediterranei* as a PHA producer is secretion of an extracellular polymeric substance (EPS) during PHA production. Simultaneous synthesis of EPS not only increases substrate demand but also adds to production cost. The C/N ratio greatly affects the EPS and PHA accumulation [82]. It was observed that increased nitrogen concentration led to enhanced EPS production whereas, nitrogen deficiency promoted PHA accumulation. The percentage of PHA yield coefficient in the sum of EPS and PHA yield coefficients at C/N ratio 5 and 35 was 51.32 and 92.8%, respectively. This indicated that most of the carbon source was converted to PHA synthesis during nitrogen limitation.

### Salinity effect

At very high salt concentration, PHA was found to be degraded faster compared to its synthesis in response to high osmotic stress when using activated sludge as inoculum (*Paracoccus* and *Thauera* as the dominant genera) [83]. In the early study of Fernandez-Castillo et al., PHA accumulation was higher at 15% salt concentration than 30% [28]. Several salt concentrations of 15, 20, 25 and 30%, were further tested on PHA accumulation in *Hfx. mediterranei*. It was clarified that the total salt (containing NaCl, MgCl_2_, MgSO_4_, CaCl_2_, KCl, NaHCO_3_ and NaBr) at a concentration of 25% was optimal for PHA accumulation [80]. Similarly, Alsafadi et al. found salt concentration of 22% to be better than 30% for PHA accumulation in *Hfx. mediterranei* [62]. Recently, salinity was found to be a parameter influencing EPS and PHA accumulation in *Hfx. mediterranei* [84]. Increasing NaCl concentration from 7.5 to 25% slightly decreased EPS production from 371.36 to 319.74 mg/g CDW, whereas PHA content increased from 46.7 to 71.1% of CDW in this strain. High salinity tends to divert carbon towards polymer storage by disrupting EPS synthesis in *Hfx. mediterranei*.

### Feeding strategy

When *Hfx. mediterranei* was cultivated in a fed-batch fermentation by feeding extruded cornstarch and yeast extract at a ratio of 1/1.7 (g/g), a CDW of 39.4 g/L containing 50.8% (wt) PHBV was obtained [85]. During this fermentation process, a pH-stat controlled feeding system was applied to maintain the pH value at 6.9–7.1. The same feeding strategy was further followed when extruded rice and extruded cornstarch at an optimized ratio of 1:8 (g/g) was fed to *Hfx. mediterranei* in repeated fed-batch fermentation [86]. A CDW of 140 g/L containing 55.6% (wt) of PHBV was obtained, indicating that the developed process was potentially more economical for semi-continuous fermentation at large-scale. *Hgn. amylolyticum* efficiently produces PHBV containing 20.1 mol% 3HV from glucose, which is the highest 3HV fraction reported among haloarchaeal species [46]. However, CDW and PHBV concentration was only 5.9 g/L and 1.5 g/L, respectively in batch mode. In order to improve PHBV productivity, fed-batch fermentation was employed where glucose was supplemented up to 10 g/L once residual glucose concentration dropped below 0.5 g/L. This fed-batch technique enhanced CDW to 29 g/L and PHBV concentration to 14 g/L [46].

#### Effect of process parameters on PHA synthesis in halophilic bacteria

Stanley et al. optimized the nutritional requirements and process parameters for PHA production using *Halomonas venusta* KT832796 in 2-L bioreactor [87]. This strain produced CDW of 3.86 g/L containing 70.56% (wt) PHB at a volumetric productivity of 0.160 g/L/h by using glucose (20 g/L) and ammonium citrate (2 g/L). A further fed-batch cultivation using four different feeding strategies was employed [87]. During carbon limitation, the pH value of medium increases due to build-up of ammonium ions [88]. Thus, the first strategy was based on pH signal where feed pump was manually switched on as pH increased above 7.0 [87]. The PHB volumetric productivity significantly increased to 0.257 g/L/h but the PHB content decreased to 39.15% (wt) as the carbon flux got diverted towards biomass synthesis yielding CDW of 87.3 g/L. In the second strategy, residual glucose concentration in the feed was controlled at 10–20 g/L. CDW of 25.7 g/L containing 58.36% (wt) of PHB was obtained with the volumetric productivity maintained at 0.156 g/L/h. In the third strategy, the residual glucose concentration was controlled at 20–50 g/L. Under this condition, PHA content of 76.67% from a CDW of 29.45 g/L was obtained. However, the PHB volumetric productivity further decreased to 0.138 g/L/h. In the fourth strategy, a high concentration of glucose close to 100 g/L was fed as a single pulse and, once utilized, feed was supplied so that the residual glucose concentration was maintained at 1–2 g/L. This strategy helped to maintain the pH value at 7.0. A net PHB content of 88.12% from a CDW of 37.9 g/L was achieved. Furthermore, PHB volumetric productivity of 0.248 g/L/h was attained, which was almost comparable to pH-signal based strategy. This work is an explicit example showing how feeding methods can enhance PHB productivity.

*Halomonas boliviensis*, can accumulate PHB when cultivated on different sugar sources. However, one of its drawbacks is the low cell density which results in low PHA productivity. Optimization of the process parameters showed that oxygen depletion in the presence of excess carbon source led to seven-fold increase in cell mass. Using this strategy, an improved CDW of 14 g/L containing 54% (wt) PHB was obtained, which was comparable with the PHA production reported by other non-halophilic producers in batch systems [89]. Additionally, in a fed-batch cultivation mode, effects of nitrogen and phosphorous sources were studied to optimize the PHB production in *H. boliviensis* [90]. NH_4_Cl and K_2_HPO_4_ were added to the medium during the initial hours of cultivation to favour cell growth. Monosodium glutamate, a cheap replacement of yeast extract, was further added for faster consumption of NH_4_Cl. After attaining cell growth, feeding of these salts were suppressed to promote PHB accumulation. This strategy led to a 44 g/L of CDW containing 81% (wt) PHB, illustrating it to be an efficient approach for obtaining high PHA production. Using the similar strategy, co-production of 42.47 g/L of PHB and 4.3 g/L of ectoine was further realized in a process comprising of two fed-batch cultures [91]. The culture condition of the first fed-batch system was conducive for high cell mass. Cells from the first fed-batch culture were harvested during the exponential phase and transferred into the second fed-batch culture. Nitrogen and phosphate supply were limited in the second fed-batch culture which favoured product formation. Ectoine was synthesized during the exponential phase whereas PHB was synthesized during the late exponential phase and stationary phase. Co-production of the two metabolites from a single fermentation unit is a promising strategy which would lower the production cost of the respective molecules. Simultaneous Saccharification and Fermentation (SSF) methodology is an energy efficient technique commonly used in the first generation biorefinery. To integrate PHA production into the biorefinery scheme, SSF technique was employed to produce PHB by *H. boliviensis* using corn mash as carbon source [92]. Interestingly, PHB production was 60% higher using SSF mode than Separate Hydrolysis and Fermentation (SHF) mode wherein corn mash was saccharified separately before fermentation. Additionally, SSF mode required less inoculum volume, single reaction vessel and moreover, saved 47% of the overall processing time. In 2015, Air-Lift Reactors (ALR) was successfully employed to produce 41% (wt) PHB from *H. boliviensis* by using starch hydrolysate and monosodium glutamate as the respective carbon and nitrogen source [93]. Although the obtained PHB content was lower compared to fed-batch reactors, ALR has the advantages of reduced construction and operational costs.

PHBV production by *H. campisalis* MCMB-1027 at different aeration and agitation in 14-L fermentor revealed that controlling dissolved oxygen is important for enhancing PHA production [94]. When dissolved oxygen was controlled within a range of 1–5% after attainment of growth phase, PHBV accumulation increased to 56.23% (wt) compared to 42.06% (wt) under uncontrolled condition. However, too low CDW of 1.33 g/L containing 49.17% (wt) PHBV was obtained when fermentation was scaled up to 120-L fermentor by keeping dissolved oxygen maintained at 1–5%.

#### Effect of fermentation apparatus on PHA synthesis

One of the exclusive advancements in PHA research is employment of open, unsterile, and continuous fermentation process for PHA production by halophiles. Application of open fermentors is an effective strategy for low-cost PHA production mainly due to reduced sterility requirement. Tan et al. carried out a 14-day unsterile fermentation of *H. bluephagenesis* TD01 using fed-batch and continuous feeding methods [51]. The process involved two fermentors. Cells collected from the first fermentor were pumped into the second fermentor for promoting PHA accumulation by maintaining nitrogen limitation in medium. 40 g/L of CDW containing 60% (wt) PHB was obtained from the first fermentor whereas 20 g/L of CDW containing 65% (wt) PHB was obtained in the second. This process aims at lowering cost and saving energy as the overall process does not involve any sterilization step and tap-water is used instead of distilled water. Moreover, use of two or more fermentors together brings up a possibility to obtain different types of PHA if different substrates are provided. Furthermore, when *Halomonas campaniensis* strain LS21 was cultured in an open and continuous sea-water based fermentation process, it could grow in a mixed substrate, whose constituent was similar to kitchen wastes, such as soluble and insoluble cellulose, proteins, fats, fatty acids, and starch [95]. During a 65-day fermentation process, a maximal CDW of 73 g/L and PHB content up to 70% (wt) were obtained without any contamination. Thus, *H. campaniensis* strain LS21 is another potential halophile for long-lasting, low-cost, and bulk production of PHB.

Other than low cost and enhanced production, efficient commercial scale fermentation also requires easy detection and determination of product formation. The methods available for PHA detection and determination are tedious and time-consuming [96]. Flow cytometry (FC) is a fully-automated and multiparametric technique for PHA determination. It rapidly and precisely studied PHB accumulation in a clear correlation with PHB concentration measured by GC-analysis in *H*. *boliviensis* [97]. FC also distinguished between two bacterial populations on the basis of presence or absence of PHB. Thus, implementations of FC in PHA production will not only allow monitoring the time for maximal productivity in a short time but will also save time for sample preparation.

## PHA metabolism and its regulation in halophiles

PHA metabolism involves its biosynthesis and degradation processes. PHA biosynthesis in halophiles has been extensively studied to reveal the synthetic pathways leading to production of different kinds of PHA [98, 99]. Three classical enzymes directly involved in PHB or PHBV synthesis include β-ketothiolase (PhaA), β-ketoacyl-CoA reductase (PhaB), and PHA synthase (PhaC). In contrast, PHA depolymerase (PhaZ) is mainly responsible for PHA mobilization. Rigorous researches have shown the immense diversity of PHA metabolic enzymes and pathways, based on the kind of halophile as well as the substrate utilized [16]. The regulation of PHA metabolism is a complex phenomenon with only a few studies reported in halophiles. Among haloarchaea, several novel proteins including PhaEC, PhaA/BktB, PhaP, PhaR, PhaZ, BdhA (3HB dehydrogenase) and PhaJ ((*R*)-specific enoyl-CoA hydratase) (Table 5), and four novel propionyl-CoA supplying pathways have been identified and characterized. In this section, we will attempt to summarize the important genes, proteins, and pathways involved in PHA metabolism and its regulation in halophiles to provide a bird’s eye view of the PHA research to the readers.

**Table 5:** Summary of key enzymes and proteins involved in PHA metabolism and regulation identified from halophiles

Process	Proteins	Organism		Features	Refs.
PHA synthesis	PhaEC	Haloarchaea	*Haloarcula marismortui*	Class III type; constitutes the active PHA synthase; co-transcription; constitutive expression; conserved lipase box-like sequence, amino acid triad (Cys-Asp-His), conserved motif of class III PHA and longer C-terminal sequence	[104]
			*Haloarcula hispanica*		[104]
			*Haloferax mediterranei*		[41]
			*Halogranum amylolyticum* TNN58	Conserved lipase box-like sequence and catalytic triad residues; 64% and 62% identity with PhaE and PhaC from *Hfx. mediterranei*	[46]
	PhaC1		*Haloferax mediterranei*	Lipase box-like sequence, conserved motif of class III PHA synthase and longer C-terminal sequence	[105]
	PhaC2			Ala instead of the last Gly in lipase box-like sequence; conserved motif not strongly conserved; longer C-terminal sequence missing, without PhaC function	[105]
	PhaC3			Lipase box-like sequence, conserved motif of class III PHA synthase and longer C-terminal sequence	[105]
	PhaC		*Halorubrum lacusprofundi*	High abundance at low temperature; C-terminal has 47% identity with the C-terminal of *Haloferax mediterranei* PhaC	[38]
	PhaC	Halophilic bacteria	*Halomonas* *elongata* DSM2581	Two candidate genes; *phaC1* is functional; unique serine instead of the first glycine in lipase box-like sequence; PhaC1 has affinity towards both 3HB and 3HV monomers	[101]
			*Halomonas* sp. O-1		
			*Halomonas* sp. R5-57	Three candidate encoding genes; the third PhaC is truncated	[106]
			*Yangia* sp. CCB-MM3	Class I type; two candidate encoding genes	[107]
	PhaC1		*Halomonas bluephagenesis* TD01	Conserved catalytic triad (Cys-Asp-His) and the conserved lipase box-like; Ser instead of first Gly in lipase box-like sequence	[52]
	PhaC2			Conserved catalytic triad (Cys-Asp-His) and the conserved lipase box-like; longer C-terminus; shorter N-terminus; Ala instead of the last Gly in lipase box-like sequence	
	BktB	Haloarchaea	*Haloferax mediterranei*	Two subunits, α and β; α is the catalytic subunit and the catalytic residues are Ser-His-His; β subunit comprises of oligo-sachharide binding domain	[109]
	PhaA				
	PhaA	Halophilic bacteria	*Halomonas elongata* BK-AG18	One subunit, catalytic residues are Cys-His-Cys	[112]
	PhaB	Haloarchaea	*Haloferax mediterranei*	NADPH-dependent; two candidate encoding genes, *phaB1* and *phaB2*; PhaB1 and PhaB2 responsible for 3HB-CoA and 3HV-CoA formation	[113]
			*Haloarcula hispanica*	NADPH-dependent; only FabG1 responsible for PHA synthesis	[114]
		Halophilic bacteria	*Halomonas bluephagenesis* TD01	NADH-dependent	[115]
PHA regulation	PhaP	Haloarchaea	*Haloferax mediterranei*	Consists of conserved amino acids and aspartate/glutamate rich regions in C-terminal; lysine acetylated	[117, 119]
		Halophilic bacteria	*Halomonas bluephagenesis* TD01	Three candidate encoding genes; only PhaP1 responsible for the amount and size of PHA granules	[116]
	PhaR	Haloarchaea	*Haloferax mediterranei*	Consists of AbrB (antibiotic resistance protein B)—like domain; regulates function of PhaP	[118]
		Halophilic bacteria	*Halomonas bluephagenesis* TD01	Regulates PHA synthesis; amphiphilic property; strong and robust emulsifier	[98, 116]
PHA degradation	PhaZh1	Haloarchaea	*Haloferax mediterranei*	Palatin-like protein; Contains classical lipase box-like	[121]
	BdhA			Encoding gene located upstream of *phaZh1*; hydrolyses 3HB monomers generated by PhaZ1 from natural PHA granules	[121]
	PhaJ			Dehydrates 3-hydroxyacyl-CoA to enoyl-CoA	[100]
	PhaZ1	Halophilic bacteria	*Halomonas bluephagenesis* TD01	Lacks signal peptide, intracellular depolymerase	[52]
	PhaZ2				
	PhaZ3			Signal peptide present, extracellular depolymerase	

### Diversity of PHA gene clusters in halophiles

The PHA-related genes are generally organized into clusters but their arrangements are highly diversified. Mostly, the PHA gene cluster is conserved within haloarchaea and organised as *maoC*-*phaR*-*phaP*-*phaE*-*phaC* [100]. Compared to this, halophilic bacteria display significant differences. *H.* *elongata* DSM2581 and *Halomonas* sp. O-1 demonstrated a genetic organization of *phaP1phaP2phaC1* where the two phasin proteins encoded by *phaP1* and *phaP2* constituted the structural proteins of the PHA granules [101]. *Halomonas* sp. SF2003 is another PHA-producing species belonging to the *Halomonadaceae* family [102]. It was noteworthy that the PHA related genes including *phaA*, *phaB*, *phaC1/phaC2*, and *phaR* were not clustered in one operon rather were located distantly apart from each other [103]. In the genome of *H. bluephagenesis* TD01, only *phaP* and *phaC1* were connected by a space of 92 bp whereas all other genes were scattered in the genome [52]. However, *phaC1* had an independent promoter which suggested that *phaP* and *phaC1* were separately transcribed driven by their own promoters. These cluster organizations might provide useful evidences about the evolution of the PHA-related genes among different groups of halophiles.

### PHA synthase

PHA synthase is the key enzyme involved in PHA biosynthesis that functions by polymerizing the hydroxyalkanoate monomers into PHA chains. In haloarchaea, PHA synthase is composed of two subunits, PhaC and PhaE, and belongs to Class III type. In contrast, PHA synthase in halophilic bacteria consists of only PhaC subunit and belongs to Class I type, which is similar to most of the PHA-accumulating bacteria. Haloarchaeal PHA synthase possesses several novel features that have been discussed in the following paragraphs.

In 2002, archaeal PHA synthase activity was detected for the first time in extremely halophilic archaeon named as strain 56 [34]. PHA synthase was covalently associated with PHB granules and its expression was found to be induced only in the condition of PHB accumulation. However, due to the lack of sequence similarity, molecular characterization of the enzymes was not possible. With the help of complete genome sequence of *Har. marismortui*, Han et al. investigated the molecular characterization of its PHA synthase genes for the first time within *Archaea* domain [104]. The annotated PhaC protein contained the highly conserved lipase box-like sequence (Gly-X-Cys-X-Gly-Gly), the amino acid triad (Cys162-Asp317-His346), and the conserved motif of class III PHA synthase (Arg-Met-Glu-X-Trp-Ile-X-Asp-X-X-Asp). These features showed its high similarity with bacterial class III PHA synthases, indicating that the annotated PhaC might be a subunit of class III PHA synthase in *Har. marismortui*. Interestingly, the protein encoded by the open reading frame immediately upstream of the *phaC* gene contained the conserved “PhaE box” and the encoded protein showed 21 to 25% identity with the PhaE subunits of bacterial class III PHA synthases. Thus, this open reading frame was designated as *phaE* gene. *Har. hispanica*, a phylogenically close archaeon to *Har. marismortui*, consisted of highly homologous *phaEC* genes and produced 9.9% (wt) of PHB [104]. Only coexpression of *phaC* and *phaE* in Δ*phaEC* mutant strain restored the PHA accumulation in *Har. hispanica*. Interestingly, unlike the haloarcheon strain 56, the PHA synthase was expressed even when *Haloarcula* cells were not cultured in PHA-accumulating medium. However, only the PhaC protein was found to be stably attached to the PHA granules whereas PhaE was not. This phenomenon indicated that PhaC and PhaE subunits constituted a novel form of class III PHA synthase in *Haloarcula* species.

Similarly, the PHA synthase of *Hfx. mediterranei* is also composed of PhaE and PhaC subunits and both the proteins constitutively expressed in nutrient-limited as well as nutrient-rich media [41]. In 2015, the PHA synthase from *Hgn. amylolyticum* TNN58 was characterized and it was reported that the *phaE* and *phaC* genes constituted an operon [46]. The amino acid sequence of PhaE and PhaC from TNN58 showed 64% and 62% identity with the counterparts from *Hfx. mediterranei*. Moreover, the PhaC subunit from TNN58 contained the conserved lipase box-like sequence (Gly-X-Cys-X-Gly-Gly) and the catalytic triad residues (Cys150-Asp305-His333). In 2017, a multi-domain protein showing 47% sequence identity in its C-terminal region with the C-terminal of *Hfx. mediterranei* PhaC was identified in *Hrr. lacusprofundi* [38]. Interestingly, this haloarchaeon is known to produce PHA at low temperatures as higher abundances of PhaC proteins were observed at low temperatures.

The molecular weight of haloarchaeal PhaE and PhaC was found to be 20 kDa and 50.1 to 58.5 kDa, respectively, which greatly differed from bacterial class III PHA synthase (40 kDa) [35]. Compared to bacterial PhaC, haloarchaeal PhaC consists of a longer C-terminal [35, 41]. This longer C-terminus is indispensable for a fully active PHA synthase as its truncation led to less accumulation of PHA. The longer C-terminal was found when PHA synthase in 18 other strains belonging to 12 haloarchaeal genera was investigated [35]. In addition to the catalytic triad (C162-D317-H346), haloarchaeal PhaC subunit also possesses two additional residues (C143 and C190), important for its enzymatic activity [35]. Moreover, unlike bacterial PhaE subunit, PhaE box was only present in *Haloarcula* and *Halorhabdus utahensis* DSM 12940, indicating that this box is not so conserved in haloarchaeal PhaEs. The alignment and phylogenetic analysis of the conserved sequences of haloarchaeal and bacterial PHA synthase distinctly clustered them into two separate domains [35]. These findings clearly suggest that haloarchaeal PHA synthase constitutes a novel subtype of class III PHA synthase.

Interestingly, besides the *phaC* gene clustered with *phaE*, the whole genome sequence analysis presented three additional *phaC* paralogs (designated as *phaC1*, *phaC2* and *phaC3*) in *Hfx. mediterranei* [105]. All the PhaC proteins exhibited 43–58% identity and contained the amino acid triad (Cys-Asp-His). Similar to PhaC, PhaC1 and PhaC3 contained the lipase box-like sequence, the conserved motif for class III PHA synthase as well as the longer C-terminal sequence. However, in the case of PhaC2, lipase box-like sequence contained an “Ala” residue instead of the last “Gly” residue. Furthermore, the conserved motif was not strongly conserved and the longer C-terminal sequence was missing in PhaC2. Although the three additional genes were not transcribed during PHBV accumulation, the heterologous expression of *Hfx. mediterranei* PhaE with each of the PhaC genes in a PHA synthase gene deleted strain, *Har. hispanica* PHB-1, led to accumulation of PHBV with varied 3HV content, except PhaC2. Phylogenetic tree analysis based on the four PhaCs from *Hfx. mediterranei* and PhaCs from other haloarchaea revealed that only PhaC was closely related to other haloarchaeal PhaCs whereas the three additional PhaC might have evolved by horizontal transfer from other source and not directly from PhaC of *Hfx. mediterranei*.

In the case of halophilic bacteria, PHA synthase shows some significant differences with the haloarcheal PHA synthase. Most importantly, PHA synthase of halophilic bacteria belongs to class I type and is composed of only one subunit, PhaC. *H.* *elongata* DSM2581 and *Halomonas* sp. O-1 have two *phaC* candidate genes, *phaC1* and *phaC2* [101]. Expression of only PhaC1 in *E. coli* JM109 led to the accumulation of PHB proving *phaC1* to be the primary functional gene. Notably, the lipase box-like sequence in the *Halomonas* PhaC1 is comprised of unique serine instead of the first glycine (Ser-X-Cys-X-Gly), which is another significant difference with haloarchaeal PhaCs. Substitution of this serine with glycine did not affect PHB content. However, substitution of the central cysteine by alanine completely abolished PHB accumulation, indicating it to be the catalytic cysteine of PhaC1. Interestingly, expression of PhaC1 in a non PHA-accumulating mutant of *R. eutropha* in the presence of sodium valerate led to PHBV synthesis, suggesting the affinity of PhaC1 towards both 3HB and 3HV monomers. The genome of *Halomonas* sp. R5-57, a marine member of the *Halomonadaceae* family, has three genes annotated as *phaC* [106]. PhaC (HALO1802) showed 91% and 86% homology with PhaC1 of *H. boliviensis* and *H. campaniensis*, respectively. The second PhaC, HALO2716, differed from PhaC1 but showed 75% homology with another PhaC from *H. boliviensis*. The third PhaC encoded by loci HALO3139 and HALO3140 was truncated, generating stop codon after 67 amino acids. Recently, *Yangia* sp. CCB-MM3, isolated from soil sediment in the estuarine possessed two PHA synthase genes, *phaC1*_*Ys*_ and *phaC2*_*Ys*_, located on chromosome 1 and 2, respectively [107]. Both of them encoded 598-amino acid proteins. Similar to *Halomonas* sp., *Yangia* PHA synthase consisted of only one subunit and belonged to class I PHA synthase.

Interestingly, new PHA synthase even exists among the halophilic bacteria. The genome of *H. bluephagenesis* TD01 consisted of two putative genes encoding PhaC [52]. PhaC1 showed almost 44% and 34% sequence similarity with the PHA synthase from *R. eutropha* H16. Furthermore, its molecular mass was found to be about 70 kDa, which was within the range of classical Class I PHA synthase (61–73 kDa). However, PhaC2 from the *H. bluephagenesis* TD01 showed some differences from the classical PHA synthase. First, the molecular mass of PhaC2 and its homologues from *H. elongata* DSM 2581, *Chromohalobacter salexigens* DSM 3043, and *Aromatoleum aromaticum* EbN1 were between 81 and 92 kDa, which was higher than the classical class I PHA synthase. Second, in the phylogenetic analysis, the common ancestor for PhaC2 from strain TD01 and its homologues were distantly clustered from the well characterized PHA synthases. Third, compared to classical class I PHA synthase, PhaC2 displayed a longer C-terminus and a shorter N-terminus. Nevertheless, the conserved catalytic triad (Cys-Asp-His) and the conserved lipase box-like were recognized in both PhaC1 and PhaC2. Interestingly, instead of the traditional lipase box-like amino acids, Gly-X-Cys-X-Gly, PhaC1 contained Ser-X-Cys-X-Gly and PhaC2 contained Gly-X-Cys-X-Ala. These differences indicate the presence of new PHA synthases in the halophilic bacteria.

Understanding the biochemical properties and the underlying catalytic mechanisms of the PhaCs will definitely enhance the biotechnological applications of these halophilic strains. Microbial mats are highly diversified microbial community which commonly accumulate high quantities of PHA. Interestingly, in a recent study, Martínez-Gutiérrez et al. identified six PHA-producing strains from hypersaline microbial mats, of which three belonged to *Halomonas* species [108]. Their PCR amplicons using primers specific to *phaC* gene showed high identity values (94–97%) with class I PHA synthase of *Halomonas aestuarii.* Microbial mats are a potential source for identifying new PHA-producing strains. Thus, the genomic reports already available will be useful for comparative genome analysis of new PHA-producing halophiles.

### β-ketothiolase

During PHA synthesis, two acety-CoA molecules are condensed to one acetoacetyl-CoA molecule by the β-ketothiolase enzyme. In halophilic bacteria, β-ketothiolase is composed of a single subunit, PhaA, whereas haloarcheal β-ketothiolase is composed of two subunits. Moreover, the amino acid residues in their catalytic subunits are distinct.

Bioinformatic and genetic analysis revealed that the β-ketothiolase enzyme responsible for PHBV accumulation in *Hfx. mediterranei* was encoded by two sets of cotranscribed genes, designated as *bktBαβ* and *phaAαβ* [109]. PhaA could only produce acetoacetyl-CoA through condensation of two acetyl-CoA molecules. Interestingly, BktB catalyzed the production of 3-ketovaleryl-CoA through condensation of acetyl-coA and propionyl-coA, along with acetoacetyl CoA formation. BktBα and PhaAα were the catalytic subunits due to the presence of the thiolase domain. The catalytic residues of these subunits comprised of “Ser-His-His” in contrary to the bacterial “Cys-His-Cys”. The other subunit, i.e., BktBβ and PhaAβ, contained oligo-sachharide binding domain which are indispensable for the β-ketothiolase activity as deletion of this domain in both BktBβ and PhaAβ completely abolished PHA synthesis. However, the N- and C-terminal regions of the BktBβ and PhaAβ had differential effects on the enzymatic activity. Deletion of the C-terminal of BktBβ reduced PHA synthesis by 70% but effect was not so significant for its N-terminal deletion. Contrarily, N-terminal deletion in PhaAβ reduced PHA synthesis by 93% and C-terminal deletion led to a complete loss of PHA synthesis. Thus, C-terminal is indispensable for both the β-subunits but N-terminal is essential for only PhaAβ activity. Furthermore, construction of hybrid enzyme, i.e, BktBα-PhaAβ and PhaAα-BktBβ demonstrated that α-subunits determined the substrate specificity and β-subunits were functionally interchangeable. This can be explained by the fact that BktBα-PhaAβ could accumulate PHBV whereas PhaAα-BktBβ accumulated PHB. Thus, BktBα-PhaAβ and PhaAα-BktBβ have similar substrate specificity like BktB and PhaA, respectively. However, knockout strain of *bktBβ* could accumulate only PHB. Only by increasing the expression level of PhaAβ restored the PHBV accumulation ability. This implied that PhaAβ inherently prefers interacting with PhaAα over BktBα to form an active β-ketothiolase.

In halophilic bacteria, PhaA is a classical β-ketothiolase similar to that of bacteria. This enzyme is composed of a single subunit. A few *halomonas* species and engineered *H. bluephagenesis* TD01 have the ability to accumulate PHBV from unrelated carbon source and thus their PhaA enzyme could condense acetyl CoA or acetyl-CoA and propionyl-CoA to generate acetoacetyl-CoA or 3-ketovaleryl-CoA [56, 98, 110, 111]. The protein structure of the putative PhaA of *H. elongata* BK-AG18 showed 68.72% identity to the β-ketothiolase structure of *Ralstonia eutropha* H16. Furthermore, the residues Cys88, His348, and Cys378 were identified to be the catalytic residues of PhaA, which is similar to the bacterial β-ketothiolase but different from the haloarcheal β-ketothiolase [112].

### β-ketoacyl-CoA reductase

The generated 3-ketoacyl-CoA is further reduced to (*R*)-3-HB-CoA and/or (*R*)-3-HV-CoA by a PHA specific 3-ketoacyl-CoA reductase (PhaB). PhaB uses NAD(P)(H) as a cofactor during this process. Most of the identified PhaB enzymes use NADPH as the cofactor. The PhaB enzyme from *H*. *bluephagenesis* TD01 utilizes NADH instead of NADPH as its cofactor.

*Hfx. mediterranei* genome encodes two PhaB proteins namely, PhaB1 and PhaB2 [113]. Knocking out of *phaB1* did not affect PHBV synthesis. However, *phaB2* knockout significantly decreased PHA synthesis and the 3HV monomer fraction in the polymer. Strikingly, knockout of *phaB1* and *phaB2* in combination completely abolished PHBV synthesis. Thus, PhaB1 has no obvious function in the presence of PhaB2; whereas PhaB1 is responsible for precursor supplying when *phaB2* was knocked out. Like PhaB, FabG belongs to the short-chain dehydrogenase/reductase (SDR) superfamily. The genomes of *Har. marismortui* ATCC 43049 and *Har. hispanica* encode multiple FabG paralogs but their amino acid identity with the bacterial counterpart is quite low, ranging from 25 to 38% [114]. Under both nutrient-rich and nutrient-limiting conditions, all the *fabG* paralogs were transcribed, however, disruption of only *fabG1* abolished PHA synthesis in *Har. hispanica.* Further complementation of the *fabG1* gene restored PHA synthesis. Intriguingly, coexpression of *fabG1* and *phaEC* in *Hfx. volcanii* DS70, a non PHA-accumulating haloarchaeon, resulted in PHA accumulation. However, coexpression of other *fabG* paralogs with *phaEC* could not lead to PHA synthesis in *Hfx. volcanii* DS70. Additionally, biochemical analysis revealed that disruption of *fabG1* decreased the NADPH-dependent acetoacetyl-CoA reductase activity. Whereas, disruption of other *fabG* genes neither affected NADPH- nor NADP-dependent acetoacetyl-CoA reductase activity. Thus, this investigation unquestionably demonstrated that *fabG1* encoded the PhaB protein in *Haloarcula* genus.

Bioinformatics analysis predicted one *phaB* gene in halophilic bacterium, *H. bluephagenesis* TD01 [115, 116]. Interestingly, the NAD(P)(H) binding domain of PhaB had a glutamate which was crucial for hydrogen bond formation with hydroxyl groups on the adenine ribose of NAD(H). The specific activity of PhaB towards NADH (30.67 mU/mg) was significantly higher than NADPH (1.71 mU/mg), indicating that PhaB from *H. bluephagenesis* TD01 is NADH-dependent.

### Propionyl-CoA synthetic pathway

Among haloarchaea, *Hfx. mediterranei* is one of the most promising and versatile PHBV-producing haloarchaeon because of its ability to incorporate 3HV monomer up to 10 mol% from unrelated and cheap carbon sources. This high 3HV molar fraction is mainly due to the presence of multiple 3HV monomer supplying pathways in *Hfx. mediterranei*. *Yangia* sp. CCB-MM3 is one of the very few halophilc bacteria which can naturally produce PHBV. One 3HV monomer supplying pathway has been predicted in *Yangia* sp. CCB-MM3. Discovery of these pathways provides a novel opportunity to engineer the PHBV synthetic pathway for obtaining PHBV polymers with controlled 3HV molar fraction.

Propionyl-CoA is an important precursor for 3HV monomer generation. *Hfx. mediterranei* simultaneously utilizes four pathways for supplying propionyl-CoA during PHBV synthesis when glucose is the main carbon source: citramalate/2-oxobutyrate pathway, aspartate/2-oxobutyrate pathway, methylmalonyl-CoA pathway, and 3-hydroxypropionate pathway [99]. Both citramalate/2-oxobutyrate pathway and aspartate/2-oxobutyrate pathway transforms 2-oxobutyrate to propionyl-CoA. Citramalate/2-oxobutyrate pathway generates 2-oxobutyrate from acetyl-CoA and pyruvate while aspartate/2-oxobutyrate pathway proceeds by converting oxaloacetate-derived threonine and methionine into 2-oxobutyrate. Finally, 2-oxobutyrate generated from these two pathways is decarboxylated to propionyl-CoA. Methylmalonyl-CoA pathway converts succinyl-CoA *via* (*S*)-methylmalonyl-CoA to propionyl-CoA. The fourth pathway, 3-hydroxypropionate pathway, was a novel finding, proposed in haloarchaea for the first time. It converts acetyl-CoA and CO_2_*via* formation of 3-hydroxypropionate to propionyl-CoA. Interestingly, almost 83.6% of the 3-HV monomer composition is provided by the citramalate/2-oxobutyrate and 3-hydroxypropionate pathway. Unlike the other two pathways which begin with TCA intermediates, these pathways begin with pyruvate and acetyl-CoA and directly store their excess carbon in PHBV granules. Recently, Williams et al. reported that higher abundance of ABC transporter proteins and enzymes involved in branched chain amino acids (BCAA) synthesis were observed in *Hrr. lacusprofundi* at low temperatures. Since, degradation of BCAA or its intermediate oxoacid precursor can generate propionyl-CoA, it was hypothesized that PHA accumulation was linked to the demand for BCAA in *Hrr. lacusprofundi* [38].

*Yangia* sp. CCB-MM3 accumulates PHBV with 7 mol% 3HV from unrelated carbon source [107]. The complete set of genes encoding the enzymes of methylmalonyl-CoA pathway including methylmalonyl-CoA mutase, methylmalonyl-CoA epimerase, and methylmalonyl-CoA decarboxylase have been predicted in this strain [107]. It can be hypothesized that succinyl-CoA is isomerized to (*R*)-methylmalonyl-CoA by methylmalonyl-CoA mutase. (*R*)-methylmalonyl-CoA is converted to its *S*-form by methylmalonyl-CoA epimerase. Finally, (*S*)-methylmalonyl-CoA is decarboxylated to propionyl-CoA by methylmalonyl-CoA decarboxylase. Thus, methylmalony-CoA pathway might supply propionyl-CoA for PHBV synthesis in *Yangia* sp. CCB-MM3.

### Regulation of PHA synthesis

Formation of uniform sized PHA granules and their distribution is a well-regulated process. Two novel PHA granule associated proteins phasin (PhaP) and GAP12 (PhaR) have been identied involved in the regulation of PHA synthesis in haloarchaea [117, 118]. The two proteins are located on the PHA granules. Their encoding genes form a cluster of *maoC*-*phaR*-*phaP*-*phaE*-*phaC* in several haloarchaeal species, including *Hfx. mediterranei*, *Har. hispanica*, *Halomicrobium mukohataei*, *Har. marismortui*, *Halorhabdus tiamatea*, *Htg. turkmenica*, *Hpg. xanaduensis*, and *Hqr. Walsbyi* [117].

PhaP consists of several conserved amino acids and aspartate/glutamate rich regions in its C-terminal that might be important for their function [117]. PhaR protein consists of an AbrB (antibiotic resistance protein B)—like domain and plays an important role in regulating PHA accumulation and granule formation [118]. The *phaP* gene was cotranscribed with *phaR* but had higher abundance probably due to its higher translational efficiency or stability. Interestingly, the expression level of *phaRP* was enhanced in the presence of PHA as the activity of the *phaRP* promoter decreased by twofolds in the Δ*phaEC* and Δ*phaPEC* mutants of *Hfx. mediterranei* [121]. However, knocking out of the *phaR* gene increased the activity of the *phaRP* promoter by fourfolds in the Δ*phaRPEC* mutant, indicating that PhaR is a negative regulator of the *phaRP* operon [118]. In the case of PHA accumulation, knockout of *phaP* gene led to 30–40% decrease in PHA yield whereas knockout of both *phaR* and *phaP* genes resulted in 75–85% decrease. Surprisingly, complementation of *phaR* gene in the Δ*phaRP* mutant fully restored PHA synthesis, unlike *phaP* which could only partly restore the PHA yield. However, knockout of either *phaP* or *phaR* led to irregular formation of PHA granules. Δ*phaP* mutant strain consisted of only one large round PHA granule with diameter of approximately 1 µm compared to the multiple granules of wild strain. Whereas, Δ*phaR* produced irregular shaped small or medium sized granules. Thus, a proper amount of PhaP protein, which is regulated by PhaR, is critical for the uniform sized PHA granule formation and separation.

Additionally, lysine acetylation is another probable factor regulating PHA synthesis [119]. Lysine acetylation is an important post-translational modification of proteins that plays a crucial regulatory role in different cellular activities. Almost 17.3% of the total proteins are lysine acetylated in *Hfx. mediterranei* [119]. Lysine acetylome of *Hfx. mediterranei* revealed that some of the key enzymes of PHA biosynthesis were acetylated including PhaAα, BktBα, and PhaE subunits [119]. Deacetylation of PhaE significantly reduced PHA synthesis probably due to inefficient interaction between deacetylated PhaE and PhaC subunit on the PHA granules [120]. Additionally, the PhaP protein was also lysine acetylated [119]. Thus, lysine acetylation plays an important role in PHA biosynthesis and its regulation in *Hfx. mediterranei.*

*H. bluephagenesis* TD01 genome consists of one gene encoding PhaR and three putative *phaP* genes [52, 116]. Among the three *phaP*s, only phaP1 was directly related to the amount and size of PHA granules [116]. However, all the three phaPs were involved in PHB synthesis as their deletion led to low PHB accumulation.

### PHA mobilization and degradation

Under conditions of carbon limitation, the intracellularly accumulated PHA is hydrolyzed by PHA depolymerase and other related enzyems so that it can be utilized by the host for growth and survival. The intracellular PHBV gradually degrades when the PHA-rich cells of *Hfx. mediterranei* are transferred into a fresh medium without carbon source [121]. Strikingly, in addition to the five PHA granule associated proteins, two new proteins were observed on the PHA granules in this strain. One protein, identified as the novel intracellular PHA depolymerase PhaZh1, was a palatin-like protein and contained a classical lipase box-like sequence (Gly-Thr-Ser-Gly-Gly), critical for hydrolytic activity. Although PhaZh1 hydrolyzed PHA granules *in vitro*, knockout of its encoding gene had no effect on intracellular PHA mobilization. This indicated the presence of alternative PHA degradation pathway in *Hfx. mediterranei*. The other protein BdhA (3HB dehydrogenase) was encoded by the gene located upstream of *phaZh1* [121]. BdhA converted 3HB monomers generated by PhaZ1 from natural PHA granules into acetoacetate. *H. bluephagenesis* TD01 has three PhaZs among which only PhaZ3 probably is an extracellular depolymerase due to the presence of a signal peptide, while PhaZ1 and PhaZ2 are intracellular depolymerases because they lacked signal peptides [52].

The fifth gene (*maoC*) of the cluster *maoC*-*phaR*-*phaP*-*phaE*-*phaC* in *Hfx. mediterranei*, redesignated as *phaJ*, encodes (*R*)-specific enoyl-CoA hydratase [100]. Despite of its organization with other *pha* genes, this gene has no function in PHA synthesis as PHBV yield was unaffected by its deletion. Instead, when the PHA-rich cells were transferred into fresh medium without carbon source, this protein catalyzed the dehydration of 3-hydroxyacyl-CoA to enoyl-CoA [100]. Enoyl-CoA is an intermediate of β-oxidation pathway, implying that PHA accumulated in *Hfx. mediterranei* is degraded and mobilized through β-oxidation pathway. As a confirmation, the expressions of genes encoding 3-hydroxyacyl-CoA dehydrogenase, the key enzyme of β-oxidation pathway, were clearly up-regulated after PHA-rich cells were transferred to fresh medium. Probably, this linkage of PHA degradation to β-oxidation pathway is a common route in haloarchaea as 96% of the species possessing PhaJ also contains the full set of genes involved in β-oxidation pathway.

The series of studies involving bioinformatics analysis and molecular characterization have provided valuable insights into the divisity of PHA metabolism and regulation in haloarchaea and halophilic bacteria [100–121]. Futhermore, the above-mentioned achievements in this secion have also refined the differences between the domains of *Bacteria* and *Archaea*.

## Strategies for novel PHA synthesis and production improvement

Although over 150 different monomers have been incorporated into PHA chains under various fermentation conditions, only few are available commercially. Now, the search for PHA with novel monomer composition and mechanical properties is still on. Sometimes, simple cofeeding strategies can synthesize novel and tailored PHA. However, most of the times combined efforts of metabolic engineering and synthetic biology are needed. Another challenge limiting PHA commercialization is low efficiency in microbial PHA synthesis. The following section is a summary on the various strategies developed to improve PHA yield and synthesize novel PHA in halophiles.

### Halomonas species

### PHBV synthesis

*H. bluephagenesis* TD01 is a promising PHA-producing halophile due to its ability to undergo fermentation under continuous unsterile process without contamination. However, it inherently produces only PHB. Analysis of the PHBV synthesis pathway in *H. bluephagenesis* TD01 revealed that propionic acid is converted into propionyl-CoA, which further enters the methylcitric acid cycle (MCC cycle) and TCA cycle [98]. The key enzyme responsible for driving propionyl-CoA to MCC cycle is 2-methylcitrate synthase encoded by *prpC* gene. The knockout strain of *prpC* gene, named as TD04, produced PHBV with 12.03 mol% of 3HV when 0.5 g/L of propionic acid was co-fed with glucose. In contrast, the wild-type strain, TD01, could incorporate only 0.82 mol% of 3HV under the same condition. Moreover, the conversion efficiency of propionic acid to 3HV underwent ninefold increase in TD04. The 2-methylcitrate synthase is an important control point for PHBV synthesis in *Halomonas* TD01 and was further confirmed by complementation of the *prpC* gene. Complementation of the *prpC* gene in TD04 reverted the 3HV level to that of TD01. Another mutant strain, named as TD08, was further developed by deletion of three *phaZ* genes along with *prpC* gene and subjected to open fermentation process performed in 500-L fermentor for 70 h. The mutant TD08 grew up to 80 g/L consisting of 70% (wt) PHBV containing 8 mol% 3HV when supplying 0.5 g/L of propionic acid and glucose concentration maintained at 20 g/L during growth phase. In contrast, this mutant gave 112 g/L CDW consisting of approximately 70% (wt) PHB when only supplied with glucose.

Despite of higher 3HV molar fraction in PHBV, engineered *Halomonas* TD04 exhibited poor CDW which led to low PHBV production. In the presence of 1.0 g/L propionic acid, TD04 could attain CDW of only 3.69 g/L containing 32.42% (wt) PHBV [98]. In contrast, CDW of TD01 was 9.17 g/L containing 78.87% (wt) PHBV. CRISPRi system consists of a denatured Cas9 protein and small guide RNA (sgRNA) complex. sgRNA guides the complex to the target DNA sequence and the catalytically dead and mutated denatured Cas9 protein with DNA binding ability binds to the targeted sequence to repress its expression. As *prpC* was critical for PHBV synthesis by *H. bluephagenesis*, Tao et al. designed seven sgRNAs (TD-prpC1 to TD-prpC7) targeting different sites of the *prpC* gene to give different repressive effects [110]. For instance, 3HV content was 1.79, 4.78, 5.72, 6.44, 8.16, 11.94, and 12.15 mol% for TD-prpC1 to TD-prpC7, whereas TD01 yielded 0.89 mol% 3HV. The combination of prpC6 and prpC7 inhibition sites further improved the 3HV content to 12.7 mol%. CDW and PHBV content of all the strains were maintained within the range of 13–15 g/L and 73–80% (wt), respectively, in the presence of 1.0 g/L propionic acid. Thus, CRISPRi system is a promising strategy allowing synthesis of PHBV with varied 3HV molar fraction, without impairing cell growth.

The requirement of propionic acid as 3HV precursor added extra cost to the fermentation process. Hence, attempts were further made to produce PHBV directly from unrelated carbon sources. The oxaloacetate generated from phosphoenolpyruvate and the TCA cycle is mostly channelled to propionyl-CoA *via* threonine synthesis. It was evident that the overexpression of threonine synthesis operon, *thrACB*, and threonine dehydrogenase encoding gene, *ilvA*, in TD08 incorporated almost 4 mol% 3HV in polymer chains using glucose as sole carbon source [111]. Additionally, the engineered strain was able to synthesize PHBV with 4.03–6.12 mol% of 3HV by utilizing sucrose, maltose, fructose, and glycerol [111]. The genome of *E. coli* consists of an operon (*scpA*-*argK*-*scpB*-*scpC*), related to conversion of succinyl-CoA to propionyl-CoA. Methylmalonyl-CoA mutase (ScpA) and methylmalonyl-CoA decarboxylase (ScpB) converts succinyl-CoA to propionyl-CoA, propionyl-CoA/succinyl-CoA transferase (ScpC) converts propionyl-CoA back to succinyl-CoA and *argK* encodes a putative kinase whose exact function is unclear. When *scpA*-*argK*-*scpB* fragment was cloned and integrated into the chromosome of TD08, resulting a mutant named as TD08AB, PHBV with 7.3 mol% 3HV was obtained using glucose as sole carbon source [122]. Contrarily, TD08 (pRE112-pMB1-scpAB), harbouring the plasmid carrying *scpA* and *scpB* resulted in lower CDW but 3HV content was as high as 25 mol%. Interestingly, TD08AB showed even better performance in open fermentation process performed in 6-L fermentor compared to TD08 (pRE112-pMB1-scpAB). Both the strains produced PHBV with 11 mol% which indicated that the scale-up increased 3HV molar fraction in TD08AB but got significantly reduced in TD08 (pRE112-pMB1-scpAB). Furthermore, the cell growth was better for TD08AB, implying that chromosomal expression is a much efficient tool for genome engineering for long-lasting fermentation process. In 2019, an efficient engineered strain that could accumulate up to 25 mol% 3HV was developed using the chromosomal expression system [25]. The authors developed several strategies to achieve it. In the first strategy, the TCA cycle was engineered by deleting the genes *sdhE* and *icl* encoding succinate dehydrogenase assembly factor 2 and isocitrate lysase in TD08AB. This redirected more metabolic flux towards 3HV synthesis and resulted in 78% increase in 3HV supplying compared to TD08AB. In the second strategy, a strain named as TY194 was developed by integrating *scpAB* under the control of a P_porin_ promoter into the chromosome of TD1.0 (Δ*prpC*). Then, *sdhE* and *icl* was deleted, alone or in combination, but the recombinant strains only accumulated PHBV with 3–5 mol% 3HV. In the third strategy, TY194 (Δ*sdhE*Δ*icl*) was grown in the presence of 2 g/L sodium gluconate, obtained from starch processing industry along with 30 g/L glucose. Sodium gluconate reduced NADH/NAD^+^ ratio which could accelerate glycolytic flux and TCA cycle. 3HV content was 18 mol% for TY194 (Δ*sdhE*Δ*icl*) whereas it was less than 5 mol% in TD08AB and TY194. In the final strategy, *ppc* encoding phosphoenolpyruvate carboxylase and *vgb* encoding *Vitreoscilla* hemoglobin (VHb) under the control of strong P_porin_ promoter and eight tandemly connected *vgb* promoter P_8vgb_, respectively, was integrated into the chromosome of TY194 (Δ*sdhE*). The resulting engineered strains generated PHBV with 3HV content varying from 3–25 mol% when grown on glucose with/without gluconate. The most efficient strain was *H. bluephagenesis* TY194 (Δ*sdhE*, G7::P_porin_-ppc) which produced 6.3 g/L CDW containing 65% (wt) PHBV with 25 mol% 3HV from glucose and gluconate. The engineered strains of *H. bluephagenesis* TD01 are enlisted in Table 6.

**Table 6:** Engineered strains of *H. bluephagenesis* TD01

Engineered strain	Parent strain	Metabolic engineering strategy	Effect on PHA synthesis	Mode of study	References
TD04	TD01	Deletion of *prpC* gene	CDW of 8.9 g/L containing 70.12% (wt) of P(3HB-*co*-12.03 mol% 3HV) in presence of 0.5 g/L of propionic acid	Shake flasks	[98]
TD-prpC6prpC7	TD01	Controlled repression of *prpC* gene using CRISPRi systems	Improved CDW of 14.67 g/L containing 73.78% (wt) P(3HB-*co*-12.70 mol% 3HV) using 1.0 g/L of propionic acid as co-substrate	Shake flasks	[110]
TD08	TD04	Deletion of three *phaZ* genes	CDW of 80 g/L containing 70% (wt) of P(3HB-*co*-8 mol% 3HV) in 0.5 g/L of propionic acid	500-L fermentor	[98]
TD08 (pSEVA341-thrACBilvA)	TD08	Overexpression of *thrACB* operon and *ilvA* gene	Synthesis of PHBV with 3HV up to 6 mol% without using 3HV precursor	Shake flasks	[111]
TD08AB	TD08	Chromosomal expression of *E. coli scpA*-*argK*-*scpB* fragment	CDW of 54 g/L containing 57% (wt) P(3HB-*co*-8 mol% 3HV)	6-L fermentor	[122]
TD08AB (Δ*sdh*EΔ*icl*)	TD08AB	Deletion of *sdhE* and *icl* genes	78% increase in 3HV yield	Shake flasks	[25]
TY194(Δ*sdhE*Δ*icl*)	TD1.0(Δ*prpC*)	Integration of *scpAB* under the control of a P_porin_ promoter; deletion of *sdhE* and *icl* genes	PHBV with 18 mol% 3HV content in presence of waste gluconate and glucose	Shake flasks	[25]
TY194 (Δ*sdhE*, G7::P_porin_-ppc)	TY194 (Δ*sdhE*)	Integration of *ppc* and *vgb* genes under the control of P_porin_ and P_8vgb_ promoter	PHBV with 25 mol% 3HV from glucose and gluconate	Shake flasks	[25]
TD40	TD01	Integration of *orfZ* gene of *Clostridium kluyveri*	Block co-polymer of P3HB4HB with 16 mol% 4HB using GBL as co-substrate	1000-L fermentor	[126]
TDH4	TD01	*orfZ* expression using moderate promoter	4HB mol% similar to TD40 was achieved by consuming 17% less GBL	7-L fermentor	[127]
TDH5	TD01	*orfZ* expression using strong promoter	40% higher 4HB mol% than TD40 was achieved from GBL as co-substrate	7-L fermentor	[127]
TDH4-pCD-Δ*phaP1*	TDH4	Deletion of *phaP1* and overexpression of *minCD*	Synthesis of large P3HB4HB granules of axial length 4 µm from GBL as co-substrate	Shake flasks	[116]
TDWT-D2	TD01	Introduction of double plasmid for expression of *orfZ*, *ogdA*, *sucD*, and *4hbd* genes	Incorporation of 0.17 mol% 4HB solely from glucose	Shake flasks	[128]
TD△*gab*D2-D2	TDWT-D2	Deletion of *gabD2* and *gabD3*	Incorporation of up to 24.9 mol% 4HB	7-L fermentor	[128]
TD68-194	TDG (*gabD2 and gabD3* deleted TD01 derivative)	Chromosomal integration of the *orfZ* and *4hbd*-*sucD*-*ogdA* genes using HRCGE system	Best 4HB-producing strain with 48.2 g/L CDW containing 75% (wt) P(3HB-*co*-16 mol% 4HB) solely from glucose	7-L fermentor	[26]
TD08 (pRE112-pMB1-udhA)	TD08	Overexpression of *udhA*	CDW of 12 g/L containing 92% (wt) PHB	Shake flasks	[98]
TD01PC	TD01	Deletion of *phaC* gene and integration of the *phaC*_Re_ downstream of the porin operon	12% increase in PHB yield	Shake flasks	[133]
TDPIΔC (pSEVA341-phaC _Re_)	TD01Δ*phaC*	Plasmid expression of *phaC*_Re_ using inducible promoter	PHB content up to 81% (wt)	Shake flasks	[133]
TDPI (pBBR1-Ppolac-phaCAB)	TDPI (TD01 derivative with *lacI* gene inserted downstream of the porin operon)	Plasmid expression of *phaCAB*_Re_ using inducible promoter	Increased PHB concentration up to 7 g/L	Shake flasks	[134]
TD-HIGH	TD01	Chromosomal expression of the *phaCAB* operon	38% enhancement in PHB production	10-L fermentor	[135]
TD01 (tat-vgb)	TD01	Periplasmic expression of *vgb* with the help of the Tat peptide signal	50% improvement in CDW and PHB concentration	Shake flasks	[137]
TDHCD-R3	TDHCD-Ro (TD01 derivative with Δ*pyrF* deleted, T7-like RNA polymerase Mmp1 and *vgb* integrated into genome and harbouring mutagenesis plasmid)	Three rounds of selection in medium containing toxic metabolites	41.7% increase in CDW and 8.2% increase in PHB content	7-L fermentor	[24]
TDHCD-R3-8-3	TDHCD-R3	Plasmid expression of *phaCAB*_Re_	12.3% increase in PHB content; CDW of 90.5 g/L with 78.8% (wt) PHB	7-L fermentor	[24]

*Halomonas* sp. SF2003 was proved as a good candidate for tailor-made PHBV synthesis as it was capable of producing PHBV with tunable proportions of 3HV monomer when various quantities of valeric acid was co-fed with glucose [123]. This strain successfully incorporated 15, 23, 27 and 35 mol% of 3HV monomer into PHB chains and thus generated PHBV polymers with greatly modified morphology and mechanical properties.

### Synthesis of PHA containing 4-hydroxybutyrate (4HB) monomer

Compared to PHB and PHBV, 4HB-containing PHA has a wide variety of mechanical and physical properties. Depending on the 4HB content, polymer property changes from crystallinity to elasticity [124]. 4HB incorporation not only widens the application potentials but also improves the biodegradability of PHA [125]. Thus, synthesis of PHA with higher 4HB molar fraction is an active research area. Most microbes require 4HB precursors including γ-butyrolactone (GBL) and 1,4-butanediol to generate 4HB monomer. Unfortunately, *Halomonas* cannot synthesize 4HB monomer even from these precursors. *H. bluephagenesis* TD01 lacks 4HB-CoA transferase gene, *orfZ*, which converts 4HB to 4HB-CoA [126]. Interestingly, when *orfZ* of *Clostridium kluyveri* was integrated into the chromosome of *H. bluephagenesis* TD01, the recombinant strain TD40 produced 7.2 g/L CDW containing 62.6% (wt) P3HB4HB with 9 mol% 4HB on 20 g/L glucose and 5 g/L GBL in shake flask experiments after 48 h. When cultivated in 1-L and 7-L fermentor under non-sterile and open conditions, the strain produced 65 g/L CDW containing 53% (wt) P(3HB-*co*-11 mol% 4HB) and 72 g/L CDW containing 63% (wt) P(3HB-*co*-12.3 mol% 4HB) after 48 h, respectively. When scaled up to 1000-L fermentor vessel, 82.6 g/L CDW containing 61% (wt) P(3HB-*co*-16 mol% 4HB) was achieved after 48 h. The produced P3HB4HB was a block copolymer and thus exhibited good elasticity with an elongation at break of 1022 ± 43%. Furthermore, the recombinant strain efficiently gave 81.4 g/ L CDW containing 73.8% (wt) P(3HB-*co*-11.6 mol% 4HB) in 7.5-L fermentor when waste corn steep liquor (CSL) along with glucose and GBL was used as substrates [124]. Further scale up to 5000-L fermentor generated 99.6 g/L CDW containing 60.4% (wt) P(3HB-*co*-13.5 mol% 4HB). The reduction in P3HB4HB content was attributed to the usage of liquid CSL instead of dried CSL powder used in 7.5-L fermentor. CSL can supply rich nutrients including amino acids and vitamins and thus, can reduce the consumption of expensive raw materials. Interestingly, replacement of glucose with waste gluconate, produced 68.1 g/L CDW containing 70.6% (wt) P(3HB-*co*-12.4 mol% 4HB) in 7.5-L fermentor. Thus, use of CSL and waste gluconate is an effective approach towards cost reduction of P3HB4HB production.

However, one of the shortcomings for TD40 is the presence of only one copy of P_tac_-*orfZ*, which is insufficient to meet the industrial demands. Therefore, promoter engineering was employed to enhance the *orfZ* gene expression. The core region from -35 box to the transcription initiation site of P_porin_, was modified to generate promoters with a wide range of transcriptional strengths [127]. Recombinant strains with moderate and strong promoter strength, named as TDH4 and TDH5, respectively, gave better performances. For instance, in 7-L fermentor, TDH4 consumed 17% less GBL compared to TD40 but the 4HB incorporated was the same with TD40 (11 mol%) [126]. Whereas, TDH5 consumed the same amount of GBL as that of TD40 but 4HB content was 40% higher than TD40 indicating that TDH5 had a higher substrate to 4HB conversion rate [127]. Thus, promoter engineering efficiently increased the 4HB content of P3HB4HB in the engineered strains. Subsequently, it was observed that TDH4 cells normally contained 4 to 12 granules of P3HB4HB. When *phaP1* was deleted, TDH4 produced only one single large granule. Further, *minCD* overexpression in TDH4-Δ*phaP1* increased 4HB mol% by 14% and the resultant P3HB4HB granule had an axial length of 4 µm [116]. Unlike TDH4 cells, recombinant TDH4-pCD-ΔphaP1 cells containing larger P3HB4HB granules could be easily precipitated at a centrifugation force of 3000×*g* for 5 min.

Despite the production of P3HB4HB with higher 4HB content, use of GBL is still a drawback as it incurs additional production cost. Hence, further pathway engineering was done to produce P3HB4HB solely from glucose. P3HB4HB synthesis pathway from glucose involves 2-oxo-glutarate dehydrogenase, encoded by *ogdA*, and succinate semialdehyde dehydrogenase, encoded by *sucD*, which directs the synthesis of succinate semialdehyde (SSA) from 2-oxoglutarate and succinyl-CoA in the TCA cycle, respectively [128]. SSA is converted to 4HB by 4-hydroxybutyrate dehydrogenase encoded by *4hbd* and further to 4HB-CoA by 4HB-CoA transferase encoded by *orfZ*. Finally, 3HB-CoA and 4HB-CoA are polymerized to produce P3HB4HB by PHA synthase. Thus, one plasmid harbouring *orfZ* gene and another plasmid harbouring *ogdA*, *sucD*, and *4hbd* genes driven by a strong promoter P_porin_ were constructed [128]. This double plasmid system, termed as D2 system, was introduced into TD01. The engineered strain, named as TDWT-D2, produced P3HB4HB with only 0.17 mol% 4HB from glucose. Thus, efforts were made to further enhance the flux of 4HB synthesis. The intracellular accumulation of SSA, which is the precursor of 4HB, is toxic to cell, hence, most bacteria possesses SSA degradation pathway. Succinate semialdehyde dehydrogenase (SSADH) converts SSA into succinate to minimize the toxicity. TD01 possesses five orthologs of succinate semialdehyde dehydrogenase (SSADH) encoding gene *gabD* (*gabD1*-*gabD5*). Interestingly, deletion of *gabD2* and *gabD3* in TDWT-D2, yielding TD△*gab*D2-D2 increased 4HB molar fraction to 4.06 mol%. Fermentation conducted in 1-L bioreactor produced P(3HB-*co*-11.0 mol% 4HB). Furthermore, in a 7-L bioreactor under fed-batch conditions, 26.3 g/L CDW containing 60.5% (wt) P(3HB-*co*-17.0 mol% 4HB) was obtained. Additionally, it was possible to tune 4HB molar fraction from 13.4 to 24.9% by varying the residual glucose concentration from 5 to 20 g/L. Thus, this is an explicit design that two interrelated pathways jointly work to synthesize P3HB4HB directly from glucose.

Very recently, fine tuning for P3HB4HB synthesis has been possible through high-resolution control of gene expressions (HRCGE) system. Gene deletions and overexpression usually lead to instability of cellular system. For instance, high accumulation of 4HB and SSA are toxic to the cell growth, but they are important intermediates for 4HB synthesis pathway. Thus, HRCGE system provides an accurate, controlled and optimized transcriptional level of gene expression by employing two inducible systems with high- and low-dynamic ranges. By using HRCGE system, the expression of *orfZ* and *4hbd*-*sucD*-*ogdA* was fine-tuned by optimizing IPTG induction concentration and varying promoter combinations [26]. This fine-tuned 4HB pathway when integrated into the chromosome of TDG (*gabD2 and gabD3* deleted TD01 derivative), generated the recombinant strain TD68-194. Compared to TD△*gab*D2-D2 [128], TD68-194 displayed improved P3HB4HB synthesis producing 48.2 g/L CDW containing 75% (wt) P(3HB-*co*-16 mol% 4HB) from glucose in 7-L fermentor [26]. Till now, this is the best strain reported to produce P3HB4HB containing higher that 15 mol% 4HB from glucose as the sole carbon source. The engineered strains derived from *H. bluephagenesis* TD01 are enlisted in Table 6.

### Enhancement of PHA synthesis

The reduction of acetoacetyl-CoA to 3-hydroxybutyrate-CoA by PhaB requires NAD(P)(H) as cofactor. *H. bluephagenesis* TD01 possesses NADH-dependent PhaB, which means it can accumulate PHA from acetyl-CoA by using NADH instead of NADPH as cofactor. Oxygen limitation significantly increases NADH/NAD^+^ ratio which indicates that the surplus amount of NADH produced during glycolysis and pyruvate metabolism can be used for PHA synthesis in *H. bluephagenesis* TD01 [115]. The well-designed study by Ling et al., proved that NADH/NAD^+^ ratio is an important parameter to channelize more acetyl-CoA towards PHA synthesis [115]. NADH/NAD^+^ ratio was more than 1.5 when *H. bluephagenesis* TD01 grows in LB medium and is higher than many other microorganisms. The genome of TD01 has the genes encoding acetate utilization proteins, including acetyl-CoA synthetase, acetate kinase, and phosphate acetyltransferase. These enzymes are expected to convert acetate to acetyl-CoA and thus generate 3HB-CoA for PHA synthesis under conditions of oxygen limitation. Co-feeding 6 g/L acetate with 30 g/L glucose generated more acetyl-CoA and enhanced PHB concentration from 8.06 to 10.4 g/L in TD01 [115]. Furthermore, manipulation of the NADH utilization pathway was another possible way to engineer the NADH/NAD^+^ ratio. Bioinformatics analysis revealed two sets of electron transfer flavoproteins (ETF-X) and one ETF-oxidoreduacase (ETF-QO) involved in the NADH-dependent respiratory chain of TD01 [115]. Both ETFs are composed of two subunits, α and β, in a same operon. ETF-X plays an electron shuttling role by transferring the reducing equivalents to the respiratory chain. Interestingly, in the presence of 3 g/L acetate, deletion of the *etf*-*x*-*β* gene enabled the mutant to accumulate larger sized PHB granule, yielding 94% PHA content of the CDW, without affecting cell growth. Besides 3HB-CoA monomer generation, acetate co-feeding positively affected the 3HV-CoA and 4HB-CoA monomers synthesis. The 3HV and 4HB monomers are derived from TCA and glyoxylate cycles. Thus, modulating NADH/NAD^+^ ratio will control the acetyl-CoA flux to these cycles. The 3HV content of PHBV in the engineered TD08AB increased from 4.2 to 8.4 mol% when 2 g/L of acetate was co-fed with glucose. Whereas, 4HB content in the engineered TDΔ*gab*D2-D2 increased from 7.9 to 12.0 mol% by cofeeding 4 g/L of acetate with glucose. Engineering the redox potential under oxygen limitation is an energy saving strategy that can reduce scale up complexities.

PntAB and UdhA are the two isoforms of pyridine nucleotide transhydrogenase that catalyzes the reduction of NADP^+^ and reoxidation of NADPH, respectively. However, it is speculated that these enzymes have important roles in maintaining the NADPH availability in the cell [129]. For instance, when there are circumstances of significant NADPH drain (such as during PHA synthesis), *udhA* overexpression reduces the metabolic burden of the cell by favouring NADPH production [130]. Overexpression of *udhA* has been found to be an effective strategy to increase product formation in various organisms including *E. coli* and *Bacillus substilis* [131, 132]. Interestingly, *udhA* overexpression in strain TD08 increased the PHB accumulation from 87 to 92% (wt) and glucose conversion to PHB from 30 to 42% [98]. Porin, a major outer membrane protein, is one of the most strongly expressed proteins in *H. bluephagenesis*. Integration of the *phaC* gene from *R. eutropha* into the chromosome of *phaC*-deletion strain of TD01, just downstream of the porin operon (recombinant strain named TD01PC), produced 9.16 g/L PHB. This yield was 12% higher than that of TD01, inferring that chromosomal expression driven by a strong promoter is a promising platform to obtain enhanced product yield [133]. Subsequently, the core promoter region of porin was identified and the *lacI* gene was integrated downstream of porin ORF, generating the strain TDPI [133, 134]. An inducible promoter was then produced by inserting the *lac* operator downstream of P_porin_ to obtain tuned gene expression. Transformation of the plasmid harbouring *phaC* gene from *R. eutropha* under the control of this inducible promoter into TDPIΔ*phaC* synthesized PHB with content varying from 2 to 81% (wt) depending on the IPTG (inducer) concentration [133]. Similarly, inducible expression of *phaCAB* operon from *R. eutropha* in TDPI yielded PHB with concentrations from 2 to 7 g/L [134]. Thus, inducible expression system effectively promoted PHB synthesis in *H. bluephagenesis*. A phage derived novel T7-like MmP1 RNA polymerase-based system was further developed which enabled efficient chromosomal expression of *phaCAB* operon. Chromosomal expression of the *phaCAB* operon in TD01 generated a new recombinant strain named TD-HIGH [135]. In a 10-L fermentor, TD-HIGH generated 69 g/L PHB compared to 50 g/L PHB produced by TD01.

Several factors including cell size, PHA granule size, oxygen availability to cell, and cell density are directly correlated to high efficiency of PHA production. Cell size is an important parameter that affects PHA granule size. When TD08 mutant was transformed with an inducible expression vector harbouring a cell division inhibitor gene, *minCD*, yielded CDW of 8.33 g/L containing 77.16% (wt) PHBV [111]. Addition of IPTG at the stationary phase of cell growth induced overexpression of the *minCD* gene and cell elongation which resulted in a CDW of 8.46 g/L containing 82.04% (wt) PHB. Highly efficient expression of *minCD* using the T7-like MmP1 system further led to 100-folds increase in cell length [135]. Increase in cell size not only enhanced PHA accumulation but also produced larger PHA granules which were easy to precipitate and recover during downstream processing. Shen et al. initially tried to increase PHA granule size by deleting *phaP* genes [116]. The three putative *phaP* genes, *phaP1*, *phaP2*, and *phaP3*, in TD01 were deleted to generate single, double, and triple knockouts. Among them, *phaP1* was majorly related to the granule size as its deletion resulted in double sized single granule formation whereas its complementation restored the multiple small sized granule formation. However, the cell sizes of the mutants were not significantly changed compared to TD01. Interestingly, *phaP1* deletion in TD01 with MinCD overexpression using T7-like MmP1, whose granule diameter was otherwise around 1 µm, produced super large PHB granules with an axial length of 10 μm. Thus, PHA granule sizes depended on the cell size. An enlarged and elongated cell will yield large and elongated PHA granules.

Oxygen availability to the cell during fermentation is another parameter that strongly affects cellular metabolism. Expression of *vgb* gene in engineered strains has allowed improvements of bioproduct formation by enhancing oxygen availability [136]. When *vgb* along with *phaCAB* under the control of P_8*vgb*_ promoter was heterologously expressed in TD01, 75% (wt) PHB was achieved compared to 36.6% (wt) PHB of the control strain containing empty plasmid under low-oxygen conditions [137]. To further enhance the oxygen uptake, a chromosomal expression system of TD01 for periplasmic expression of *vgb* with the help of the twin-arginine translocation (Tat) peptide signal was constructed. Periplasmic *vgb* expression resulted in 50% improvement of CDW and PHB concentration. This strategy enhanced ATP and NADH/NAD^+^ levels in *H. bluephagenesis* and improved its cell density and PHB synthesis.

Next, a high-cell-density strain was obtained by genome-wide random mutagenesis and selection stress method to enhance PHB synthesis [24]. Firstly, an engineered strain was obtained by *pyrF* gene knockout and T7-like RNA polymerase Mmp1 gene and *vgb* gene integration into the genome of TD01. The resulting strain was further transformed by an expression plasmid containing *pyrF* in the backbone, to ensure long term selection process, and an error-prone DNA polymerase III ε subunit from TD01. The finally obtained strain, named as TDHCD-Ro, was allowed to grow for a few generations in selective medium containing toxic metabolites including acetate, formate, lactate, and ethanol. After 3 selection rounds, the best mutant strain was selected by determining its CDW and PHA content. The finally obtained mutant, TDHCD-R3, showed 41.7% increase in CDW and 8.2% increase in PHB content compared to TDHCD-Ro. Construction of this high-cell-density strain was based on the assumption that the strain which survived in the presence of toxic metabolites would have high cell density. Thus, the recombinant strain TDHCD-R3 was used to give further enhanced PHB content. The plasmid harbouring *R. eutropha phaCAB* operon with suitable promoter and ribosome binding site (RBS) sequence was expressed in TDHCD-R3. The resulting strain TDHCD-R3-8-3 showed a 12.3% increase in PHB content compared to TDHCD-R3. In a 7-L fermentor, TDHCD-R3-8-3 attained 90.5 g/L CDW with 78.8% (wt) PHB content while TD01 grew up to 80.6 g/L CDW with 69.4% (wt) PHB content. Interestingly, for a same cultivation time, glucose consumption of TD01 was 253.5 g more than TDHCD-R3-8-3, indicating a higher conversion efficiency of the latter strain.

Among *Halomonas* species, *H.* *campaniensis* LS21 is another promising species that can accumulate PHB under open and unsterile fermentation conditions. To further improve its production efficiency, cell morphology related genes *mreB* and *ftsZ* were deleted [138]. Since they are essential genes, a temperature-responsible plasmid system was developed to compensate the expression of *mreB* or *ftsZ* in the disrupted mutants. The plasmids replicated at 30 °C so that the mutants attained certain cell density, and then at 37 °C the plasmids failed to replicate and were eliminated resulting in controlled cell expansion or elongation. In an unsterile 7.5-L bioreactor, the wild type grew up to 27 g/L CDW containing 30% (wt) PHB, whereas both Δ*mreB* mutant and Δ*ftsZ* mutant grew up to 34 g/L containing 51% and 60% (wt) PHB, respectively. Thus, controlling cell morphology efficiently enhanced PHB accumulation in *H.* *campaniensis* LS21. Furthermore, a self-flocculating *H. campaniensis* strain LSKO was obtained by knocking out a nonessential *etf* operon encoding the α and β subunits of an electron transfer flavoprotein [139]. LSKO accumulated up to 45% (wt) PHB. RBS-optimized *phaCAB* operon from *C. necator* under the control of P_porin_ was transformed to LSKO to get another strain named LSKO-H36-6. LSKO-H36-6 accumulated 85% (wt) PHB and granules occupied the entire intracellular space. This was also an innovative approach to reduce energy consumption and wastewater generation as the flocculating cells could precipitate without the need of centrifugation and the fermentation supernatant could be efficiently reused in the next cycle.

### Haloarchaea

Among Haloarchaea, *Hfx. mediterranei* is a potential cell factory for PHA synthesis. One of the most important advantages of *Hfx. mediterranei* is its ability to produce PHBV directly from cheap unrelated carbon source. Moreover, haloarchaea-produced PHBV exhibits distinctive features like lower melting point, lower polydiseperse index, and better hemocompatibility performance compared to bacteria-produced PHBV [4, 140]. The following section will summarize the efforts made to develop *Hfx. mediterranei* as one of the most successful haloarchaea for PHA synthesis.

### High-level PHBV synthesis

EPS synthesized by *Hfx. mediterranei* gives it a slimy appearance which helps them to aggregate during extreme conditions. However, EPS synthesis presents several disadvantages to PHA synthesis. First, the secreted EPS increases viscosity of the medium which decreases dissolved oxygen. Second, viscosity increases foaming and thus addition of anti-foaming agent is needed. Third, the carbon source gets divided between EPS and PHA synthesis pathways. In total, all these factors reduce PHA yield, increase fermentation cost, and make PHA recovery difficult. Whole genome sequencing of *Hfx. mediterranei* identified the gene cluster involved in EPS synthesis [141]. Knocking out of the gene cluster abolished EPS synthesis and generated a strain named ES1. Interestingly, PHBV production for ES1 was 20% higher than the wild type and the viscosity of the culture significantly reduced. Moreover, sugar consumption rate and requirement of anti-foaming agent decreased which can reduce the fermentation cost. Thus, ES1 is a highly efficient and promising platform for synthesis of novel PHA. Very recently, another high-yielding ES1 derived strain was serendipitously developed while investigating the phosphoenolpyruvate (PEP)/pyruvate interconversion of *Hfx. mediterranei* [142]. Surprisingly, deletion of a phosphoenolpyruvate synthetase-like (*pps*-*like*) gene accumulated more intracellular PHBV granules and also significantly increased the PHBV content by 70.46% in shake flask fermentation. Preliminary results explained that *pps*-*like* gene knockout upregulated the genes responsible for glucose transportation and PHBV monomer generation. Moreover, the *pps*-*like* gene knockout significantly upregulated the PHA synthesis and regulation gene cluster. Their high expression level probably enhanced PHBV accumulation in *Hfx. mediterranei*.

### Synthesis of PHBV with higher 3HV contents

Han et al. evaluated the effect of valerate supplementation on PHBV synthesis by ES1 and obtained tailor-made random PHBV (R-PHBV) and higher-order copolymers (O-PHBV) [4]. Co-feeding of valerate along with glucose yielded PHBV with 3HV molar fraction ranging from 9-57 mol%. Compared to the random arrangement of 3HB and 3HV in R-PHBV, O-PHBV consisted of block segments of PHB and PHV and random segments of PHBV. The blocky segments of O-PHBV enhanced its crystallinity and Young’s modulus. Additionally, O-PHBV copolymer displayed a novel foveolar cluster-like surface morphology with high surface roughness and high hydrophobicity. Most interestingly, O-PHBV exhibited 3.5 times higher platelet adhesion and 5 times shorter coagulation time than R-PHBV. Thus, synthesis of O-PHBV with excellent hemostatic property is a novel strategy that can provide more biopolymers with great potential in biomedical fields. Furthermore, as 3HV content increased, the PHBV copolymers became more elastic, with elongation at break ranging from 5 to 430%. These polymers have extremely low endotoxin concentrations due to the structure of archaea cell envelopes, and avoid acute inflammatory responses. Also, they exhibit diversified ranges of biodegradation behaviour when implanted in the rabbit dorsal subcutis [5]. This implied that the novel PHA produced by *Hfx. mediterranei* has immense potentials in biomedical applications. Feeding of VFA as sole carbon source can produce PHBV with controlled composition in *Hfx. mediterranei* [143]. The carbon number of the VFA greatly influenced the monomer composition as carbon-even generated 3HB rich polymer whereas carbon-odd produced 3HV rich polymer. Mixtures of butanoic acid and pentanoic acid synthesised bespoke random, block, and blend PHBV with 3HV content varying from 1 to 100 mol% depending on the pentanoic acid percent in the mixture. PHBV with more than 50 mol% 3HV exhibited lower melting temperature and a wide of range of processing temperature. Compared to the blend PHBV, block and random PHBV exhibited desirable thermal stability. Thus, VFA feeding is an effective strategy to obtain higher 3HV content in *Hfx. mediterranei.*

### Synthesis of PHA containing 4HB monomer

*Hfx. mediterranei* has been less investigated for the synthesis of PHA containing 4HB monomer. However, the most interesting fact revealed from the studies of Koller et al. is that *Hfx. mediterranei* can synthesis PHBV4HB when GBL was supplied along with valerate and whey sugars [23]. *Hfx. mediterranei* accumulated 87.5% (wt) of P(3HB-*co*-21.8 mol% 3HV-*co*-5.1 mol% 4HB) at a concentration of 14.7 g/L in a 10-L bioreactor [23]. Similarly, co-feeding of GBL with crude glycerol obtained from biodiesel industry yielded 68.5% (wt) P(3HB-*co*-10 mol% 3HV-*co*-5 mol% 4HB) at a final concentration of 11.1 g/L [68]. These findings inferred that 4HB monomer supplying pathway from GBL is present in *Hfx. mediterranei*. However, since the 4HB content is relatively low, further genetic engineering *Hfx. mediterranei* to enhance 4HB supplying is an encouraging research field.

## Conclusion and future perspectives

Extensive plastic usage is a global threat to our environment which needs to be addressed with concerted effort. Microbial synthesis of bioplastic PHA and its application is an attractive solution to avert this problem. Compared to other extremophiles, halophiles have been well explored in terms of their biotechnological process in PHA synthesis. They have already been considered as a promising cell factory for PHA synthesis due to their inherent advantages. In this review paper, we have elaborated four major aspects of PHA research: mining of PHA-accumulating halophiles, strategies to reduce the cost of PHA synthesis, PHA metabolism and its regulation in halophiles, and finally, metabolic engineering of halophiles for enhancing PHA production and novel PHA synthesis. Although PHB is more commercialized, enhanced synthesis of PHBV with varied 3HV content has been materialized with advanced metabolic engineering tools and approaches. Tailor-made PHBV synthesis with desired properties has already been accomplished. Furthermore, 4HB monomer synthesis and incorporation into PHA chains by halophile is a milestone in this field.

Dissection of PHA metabolic pathways and its regulation in halophiles is an important avenue in biotechnology. The genetic manipulation and pathway engineering have provided various alternative routes to enhance monomer flux for enhanced scl-PHA synthesis. All the extensive research work carried out in halophiles can speed up their metabolic engineering to realize PHA commercial production at low cost. Moreover, availability of the detailed genetic information on various *Halomonas* species and haloarchaeal species shall definitely facilitate high PHA production from more species. Synthesis of novel PHA is a highly demanding research area as each type of newly synthesized polymer shall exhibit unique property for application which will elevate the status of bioplastics in near future. The heterologous construction of 4HB pathway in *H. bluephagenesis* is not only an excellent approach to generate 4HB monomer but also it provides research directives for 4HB synthesis in more and more halophilic strains. Continued efforts and further investigation with the metabolic engineering tools and strategies are needed to achieve tailor-made 4HB containing PHA.

## Data Availability

Not applicable.

## Abbreviations

PHA: Polyhydroxyalkanoates
CDW: Cell dry weight
3HB: 3-Hydroxybutyrate
PHB: Polyhydroxybutyrate
3HV: 3-Hydroxyvalerate
PHBV: Poly(3-hydroxybutyrate-*co*-3-hydroxyvalerate)
4HB: 4-Hydroxybutyrate
P3HB4HB: Poly(3-hydroxybutyrate-*co*-4-hydroxybutyrate)
PHBV4HB: Poly(3-hydroxybutyrate-*co*-3-hydroxyvalerate-*co*-4-hydroxybutyrate)
VFA: Volatile fatty acids
SDCLA: Seaweed derived crude levulinic acid
C/N: Carbon/nitrogen
EPS: Extracellular polymeric substance
SSF: Simultaneous saccharification and fermentation
ALR: Air-lift reactors
FC: Flow cytometry
BCAA: Branched chain amino acids
GBL: γ-Butyrolactone
HRCGE: High-resolution control of gene expressions
PPS-like: Phosphoenolpyruvate synthetase-like
